# d-Alanylation of Lipoteichoic Acids in *Streptococcus suis* Reduces Association With Leukocytes in Porcine Blood

**DOI:** 10.3389/fmicb.2022.822369

**Published:** 2022-04-18

**Authors:** Sophie Öhlmann, Ann-Kathrin Krieger, Nicolas Gisch, Marita Meurer, Nicole de Buhr, Maren von Köckritz-Blickwede, Nicole Schütze, Christoph Georg Baums

**Affiliations:** ^1^Institute of Bacteriology and Mycology, Centre for Infectious Diseases, Faculty of Veterinary Medicine, University of Leipzig, Leipzig, Germany; ^2^Division of Bioanalytical Chemistry, Priority Area Infections, Research Center Borstel, Leibniz Lung Center, Borstel, Germany; ^3^Institute for Biochemistry, University of Veterinary Medicine Hannover, Hannover, Germany; ^4^Research Center for Emerging Infections and Zoonoses (RIZ), University of Veterinary Medicine Hannover, Hannover, Germany; ^5^Institute of Immunology, Centre for Infectious Diseases, Faculty of Veterinary Medicine, University of Leipzig, Leipzig, Germany

**Keywords:** *Streptococcus suis*, IL-1β, *dltA*, monocyte, oxidative burst, ROS, complement, lipoteichoic acids

## Abstract

*Streptococcus suis* (*S. suis*) is a common swine pathogen but also poses a threat to human health in causing meningitis and severe cases of streptococcal toxic shock-like syndrome (STSLS). Therefore, it is crucial to understand how *S. suis* interacts with the host immune system during bacteremia. As *S. suis* has the ability to introduce d-alanine into its lipoteichoic acids (LTAs), we investigated the working hypothesis that cell wall modification by LTA d-alanylation influences the interaction of *S. suis* with porcine blood immune cells. We created an isogenic mutant of *S. suis* strain 10 by in-frame deletion of the d-alanine d-alanyl carrier ligase (DltA). d-alanylation of LTAs was associated with reduced phagocytosis of *S. suis* by porcine granulocytes, reduced deposition of complement factor C3 on the bacterial surface, increased hydrophobicity of streptococci, and increased resistance to cationic antimicrobial peptides (CAMPs). At the same time, survival of *S. suis* was not significantly increased by LTA d-alanylation in whole blood of conventional piglets with specific IgG. However, we found a distinct cytokine pattern as IL-1β but not tumor necrosis factor (TNF)-α levels were significantly reduced in blood infected with the Δ*dltA* mutant. In contrast to TNF-α, activation and secretion of IL-1β are inflammasome-dependent, suggesting a possible influence of LTA d-alanylation on inflammasome regulation. Especially in the absence of specific antibodies, the association of *S. suis* with porcine monocytes was reduced by d-alanylation of its LTAs. This *dltA*-dependent phenotype was also observed with a non-encapsulated *dltA* double mutant indicating that it is independent of capsular polysaccharides. High antibody levels caused high levels of *S. suis*—monocyte—association followed by inflammatory cell death and strong production of both IL-1β and TNF-α, while the influence of LTA d-alanylation of the streptococci became less visible. In summary, the results of this study expand previous findings on d-alanylation of LTAs in *S. suis* and suggest that this pathogen specifically modulates association with blood leukocytes through this modification of its surface.

## Introduction

*Streptococcus suis* (*S. suis*) is a zoonotic pathogen, mainly associated with meningitis, arthritis, and septicemia in pigs (Gottschalk and Segura, 2019), but also a frequent colonizer of the upper respiratory tract and the tonsils of healthy pigs (Baele et al., 2001). To cause invasive infections, *S. suis* needs to enter the bloodstream, survive, spread, and proliferate in different tissues. Consequently, the immune response in blood represents an important line of defense and bacteremia plays a key role in the pathogenesis of invasive *S. suis* infections. Different virulence-associated factors of *S. suis* have been proposed to support bacterial survival in blood. Especially the polysaccharide capsule plays an important role, by protecting *S. suis* against phagocytosis (Smith et al., 1999; Fittipaldi et al., 2012). Its composition is very divers, resulting in 29 serotypes (Okura et al., 2016) of which serotype 2 is the most prevalent in invasive infections (Goyette-Desjardins et al., 2014).

As in other Gram-positive bacteria, lipoteichoic acids (LTA), which are linked to the cell membrane by a lipophilic, glycosylated diacyl-glycerol anchor (Percy and Gründling, 2014) are major cell wall components of *S. suis* (Gisch et al., 2018). One common modification of these LTAs is d-alanine residues that are non-stoichiometrically bound to glycerol or ribitol moieties within the polymeric LTA chains. The four essential enzymes required for this process are encoded by the dltABCD operon (Neuhaus and Baddiley, 2003; Brown et al., 2013; Wood et al., 2018). It has been established that DltA transfers d-alanine in the cytoplasm of the cell into the carrier protein DltC (Brown et al., 2013; Reichmann et al., 2013) and that inactivation of *dltA* leads to the abrogation of alanylation of LTA in *S. suis* (Fittipaldi et al., 2008). The ability to introduce d-alanine into the poly-glycerophosphate chains of LTAs has been recognized as a protective mechanism against cationic antimicrobial peptides (CAMPs) for numerous streptococcal species (Poyart et al., 2003; Kristian et al., 2005; Kovács et al., 2006; Chan et al., 2007; Fittipaldi et al., 2008). CAMPs commonly function by crossing the peptidoglycan barrier and interacting with the anionic phospholipid of the cytoplasmic membrane. The resistance induced by LTA d-alanylation might be due to the resulting alteration of the electric charge, the rigidity, and permeability of the bacterial cell wall (Saar-Dover et al., 2012), leading to an reduced interaction with CAMPs.

An increased susceptibility to neutrophils due to a lack of LTA d-alanylation was repeatedly described for different streptococci including *S. suis* (Poyart et al., 2003; Kristian et al., 2005; Fittipaldi et al., 2008). The exact mechanisms behind this remain unclear. Neutrophils can kill bacteria by different strategies like phagocytosis and oxidative burst, the formation of neutrophil extracellular traps (NETs), or degranulation. In these mechanisms, CAMPs released from neutrophil granules can be involved in bacterial killing (Wessely-Szponder et al., 2010).

Bacterial proliferation and exacerbated inflammation during bacteremia are important steps in the pathogenesis of meningitis and septicemia, often leading to sudden death in pigs and the streptococcal toxic shock-like syndrome (STSLS) described in human patients (Tang et al., 2006). While Interleukin (IL)-1 signaling was shown to be beneficial to control and clear streptococcal burden (Lavagna et al., 2019) an exacerbated inflammatory response due to inflammasome activation is able to induce STSLS (Lin et al., 2019).

d-alanylation of LTA in pneumococci might contribute to an increased production of IL-1β (IL-1F2; Sims et al., 2001) in the upper respiratory tract of infected infant mice (Zafar et al., 2019). Also for *S. suis*, a modification of the cytokine response in dependence on the level of LTA d-alanylation was described (Lecours et al., 2011). Nevertheless, the putative role of LTA as ligand of pattern recognition receptors (PRRs) is controversially discussed (Hashimoto et al., 2006; von Aulock et al., 2007; Zähringer et al., 2008; Gisch et al., 2013, 2018). A possible immunomodulatory effect of d-alanylation might not be attributable to direct interaction of LTA with PRRs but rather to changes in the bacterial cell wall and resulting differences in bacteria-host cell association. Accordingly, we investigated the working hypothesis in this study that d-alanylation of *S. suis* LTAs modulates the interaction with leukocytes, phagocytosis, and cytokine production in infected porcine blood.

## Materials and Methods

### Bacterial Strains and Culture Conditions

*Streptococcus suis* serotype 2 strains 10 and 10cpsΔEF, a mutant deficient in capsule expression due to deletion of the genes *cps*E and *cps*F [*epf* encoding the extracellular factor (EF) is not deleted in this mutant], were kindly provided by Hilde Smith, DLO-Lelystad (Smith et al., 1999). The genome sequence of strain 10, which is part of clonal complex 1, has recently been published (Bunk et al., 2021). The strain was confirmed to be highly virulent in experimental infection of piglets leading to meningitis and other pathologies (Baums et al., 2006, 2009; Seele et al., 2013; de Buhr et al., 2014; Rungelrath et al., 2018). In this study, wild-type (wt) strain 10 and its non-encapsulated mutant 10cpsΔEF were used to generate the two isogenic knock out mutants 10Δ*dltA* and 10cpsΔEFΔ*dltA*, respectively.

Unless otherwise indicated, *S. suis* strains were cultured in Todd-Hewitt broth (THB, Becton Dickinson, 249240) or on Columbia agar plates with 5% sheep blood (Oxoid, PB5039A) at 37°C and 5% CO_2_. *Escherichia coli* (*E. coli*) DH5α was grown in lysogeny broth (LB, Carl Roth, X968.1) at 37°C under constant shaking or on LB agar plates. When needed, chloramphenicol (Cm, Carl Roth, 3886.2) was added at a concentration of 3.5 μg/ml for *S. suis* and 8 μg/ml for *E. coli*.

### DNA Techniques and Primers

Restriction enzymes (BamHI R0136, SalI R0138, PstI R0140, CIP M0525, and AccI R0161), ligase (ElectroLigase® M0369), and polymerases (One*Taq®* M0480, Phusion® M0530) were purchased from New England Biolabs and used according to manufacturer’s recommendations. Standard DNA manipulations were performed as described (Green and Sambrook, 2012). Chromosomal DNA of *S. suis* strain 10 served as a template for all PCRs conducted for generation of inserts. Oligonucleotide primers were designed based on the sequence of SSU0596 in the genome of *S. suis* P1/7.^1^ This nucleotide sequence is also found without any difference in the recently published genome of strain 10 (SSU10_RS03070; Bunk et al., 2021) as well as in the 2020 published genome of S10 (van der Putten et al., 2020).

### Targeted Mutagenesis of *dltA* in *Streptococcus suis* Strain 10 and 10cpsΔEF

The thermosensitive shuttle vector pSET5s (Takamatsu et al., 2001) was used as a backbone to generate the plasmid pSET5Δ*dltA* enabling allelic exchange of *dltA* as follows:

DNA fragments corresponding to regions upstream and downstream of the *dltA* gene were amplified using primers dltA_FrA_for_BamHI (TAAGGATCCTCACATTTTTTGCGA ATG) and dltA_FrA_rev_SalI (ATGGTCGACCGCTCTAAC ATACTGCTAA) as well as dltA_FrB_for_SalI (TCCGTCGACTG CAAATGGGAAGATTG) and dltA_FrB_rev_PstI (GAT CTGCAGGGCCACTCGAAATAGTTG). The fragments were digested with the restriction enzymes indicated in the names of the primers and inserted in the multiple cloning site of pSET5s.

Restriction analysis and sequencing were carried out to verify the sequence of the resulting plasmid prior to transformation into competent *S. suis* strain 10, whereby competence was induced using a synthetic peptide (H-GNWGTWVEE-OH, jpt) as described before (Zaccaria et al., 2014).

The isogenic mutant 10Δ*dltA* was verified by PCR, sequencing of the deletion site and non-radioactive Southern blot analysis as described before (Rungelrath et al., 2018). Chromosomal DNA was digested by AccI and primer pairs used for generation of the probes were dltA_Ssuis_for (5′-TATGTATTGGGCTCCGA CGCTTG-3′) plus dltA_Ssuis_rev (5′-AAGTTGGCGAGTCTGG TTTGG-3′) to verify the absence of the *dltA* gene and pSET5sSondefor (5′-CGAAAAAAAGAGTTATGATTTCTCTG -3′) plus pSET5sSonderev (5′-GGTTTTTTATAGTGCTTTCCA TTTTG-3′) to confirm complete excision of the plasmid from the bacterial chromosome.

To construct a 10cpsΔEFΔ*dltA* double mutant, we transformed the plasmid pSET5Δ*dltA* into the 10cpsΔEF mutant and followed the mutagenesis protocol described above.

### Extraction and Isolation of LTA

Bacteria were grown in THB to an optical density at 600 nm (OD_600_) of 1 and harvested by centrifugation at 5,000 × *g*, 4°C for 15 min. LTA isolation and purification were performed as described elsewhere (Heß et al., 2017). Yields of LTA preparations from 5 L of bacterial culture were as follows: strain 10, 8.7 mg; strain 10Δ*dltA*, 3.9 mg.

### Nuclear Magnetic Resonance Spectroscopy

Deuterated solvents were purchased from Deutero GmbH (Kastellaun, Germany). Nuclear magnetic resonance (NMR) spectroscopic measurements were performed in deuterated 25 mM sodium phosphate buffer (pH 5.5; to suppress fast de-alanylation) at 300 K on a Bruker Avance^III^ 700 MHz (equipped with an inverse 5-mm quadruple-resonance Z-grad cryoprobe) as described (Gisch et al., 2018).

### Extraction of mRNA and qRT-PCR

Bacterial RNA was extracted from exponential-phase THB cultures (OD_600_ of 0.5) of the different *S. suis* strains used in this study. In detail, the pellet of a 10-ml culture (centrifugation: 10 min, 2,600 × *g*, 4°C) was resuspended in 1 ml ice-cold Trizol (Sigma T9424) and stored at −80°C until RNA extraction. Isolation was conducted as previously described (Willenborg et al., 2011). Concentration and purity of the isolated RNA were determined using the Agilent 2100 bioanalyzer (RNA 6000 Pico kit; Agilent Technologies Inc., Santa Clara, CA, United States). Quantitative real-time PCR (qRT-PCR) of reverse-transcribed RNA was designed to analyze the expression of *dltA*, *dltC—dltD*, SSU10_RS03090 (low temperature requirement protein A, downstream of *dltD*), SSU10_RS03040 (glucosamin-6-phosphate deaminase, upstream of *dltA*), and the housekeeping gene *gyrB*. The respective primers are listed in Supplementary Table S1. qRT-PCR was conducted with the AriaMX real-time PCR system (Agilent Technologies Inc., Santa Clara, CA, United States), as previously described (Willenborg et al., 2011). The following modified program was used as: an initial denaturation step at 95°C for 15 min and 40 cycles of denaturation at 94°C for 15 s, annealing at 60°C for 30 s, and amplification at 72°C for 30 s. As negative controls, we included (i) water, (ii) a no-template control, and (iii) no reverse transcriptase control to exclude false positive results due to contamination with DNA. Products were verified by melting-curve analysis and 1.5% agarose gel electrophoresis.

### Cytochrome C Binding Assay

The assay was essentially performed as previously described (Kristian et al., 2005), except that bacteria were adjusted to an OD_600_ of 1 in morpholinepropanesulfonic acid (MOPS) buffer (20 mM, pH 7) before adding 0.5 mg/ml cytochrome C for 10 min at room temperature. After centrifugation (14,000 × *g*, 3 min, room temperature), the cytochrome C content of the supernatant was quantified photometrically at 530 nm (OD_B_). MOPS buffer containing 0.5 mg/ml cytochrome C was incubated under the same conditions without bacteria as a control (OD_A_). The percentage of bound cytochrome C was calculated as follows:


$$\mathrm{Bound cytochrome}\;C\;\left(\%\right)=\left[{\mathrm{OD}}_{A}–{\mathrm{OD}}_{B}/{\mathrm{OD}}_{A}\right]\times 100$$

### Microbial Adhesion to Hydrocarbons Assay

Hydrophobicity of *S. suis* was evaluated by measuring bacterial adhesion to hexadecane (Sigma, H6703) following a previously described protocol (Srikham et al., 2021) with slight modifications. Briefly, *S. suis* strains were cultured overnight and harvested by centrifugation (3,900 × *g*, 10 min, 4°C). Pellets were resuspended in PBS and washed twice before adjusting the suspensions to an OD_600_ of 1 (OD_A_). Then, 2 ml of bacterial suspension was mixed with 400 μl of hexadecane and tubes were vortexed for 30 s. The mixture was allowed to separate into two phases for 30 min at room temperature. The aqueous phase was collected and OD_600_ (OD_B_) was measured. Cell surface hydrophobicity was calculated as follows:


$$\mathrm{Hydrophobicity}\;\left(\%\right)=\left[1–\left({\mathrm{OD}}_{B}/{\mathrm{OD}}_{A}\right)\right]\times 100$$

### Antimicrobial Susceptibility

The cathelicidin PR-39 (RRRPRPPYLPRPRPPPFFPPRLPPRIP PGFPPRFPPRFP) was kindly provided by Ralf Hoffmann from the Institute of Bioanalytical Chemistry of the University of Leipzig. Bacitracin was obtained from Carl Roth (5655.1), colistin, and polymyxin B were obtained from Sigma (C5561 and P4932). Minimal bactericidal concentrations (MBC) were defined as the lowest concentration of the antimicrobial agent where no bacterial growth occurred. To determine the MBC, we followed a previously described protocol for evaluation of minimal inhibitory concentrations (MIC; Fittipaldi et al., 2008) and additionally plated aliquots of the suspensions on blood agar plates after the incubation period of 24 h at 37°C. While *S. suis* was cultured in 100 μl THB with serial dilutions of bacitracin, colistin, and polymyxin B as described in the above mentioned protocol, evaluation of PR-39 MBC was performed in 50 μl Rosewell Park Memorial Institute (RPMI) 1640 (Gibco, 11835063) containing 5% cation-adjusted Mueller Hinton broth (CA-MHB, Oxoid CM0405, MgCl_2_.6H_2_O Sigma M2670, and CaCl_2_.2H_2_O Merck 2382) based on the finding that MICs measured in this medium resembled MICs determined in cerebrospinal fluid of pigs (Meurer et al., 2019). Each assay was performed in triplicates and repeated at least three times.

### Bactericidal Assays in Whole Blood

Comparative analysis of survival of *S. suis* 10 and its mutants in heparinized porcine blood was conducted multiple times in samples drawn from 8-week-old piglets originating from different commercial pig farms. Collection of blood was approved by the state Saxony, Germany, under the permit numbers TVV 57-18 and A09/19. The assay was conducted as described before (Seele et al., 2013) with an increased infection dose of 1 × 10^7^ CFU/ml. Survival factors (SF) were determined by dividing the CFUs after the indicated incubation time by the CFU value at time zero.

### Far Red Labeling of *Streptococcus suis*

Stocks of *S. suis*, labeled with CellTrace Far Red fluorescent dye (Thermo Fisher Scientific, C34564; S. suis*FR), were generated using exponential phase THB cultures (OD_600_ 0.5). Bacteria were harvested from 8 ml of these cultures (2,500 × *g*, 10 min, 4°C) and washed twice with PBS before resuspending the pellet in 1 ml PBS and adding 1 μl of FR stock solution (1 mM in DMSO). After an incubation for 20 min at 37°C under rotation in the dark, bacteria where washed again with PBS and finally resuspended in 1 ml THB containing 15% glycerol. Aliquots were frozen in liquid nitrogen. Unlabeled stocks were treated the same way without addition of FR.

### Combined Oxidative Burst and Granulocyte Association Assay

Measurement of oxidative burst and the association of *S. suis* with porcine granulocytes was essentially conducted as described before (Rungelrath et al., 2020), except that whole blood samples were used. Briefly, S. suis*FR stocks were added to 100 μl whole blood of 8-week-old piglets at a concentration of 10^7^ CFU/ml. After 15 min of incubation at 37°C, dihydrorhodamine123 (DHR123, Sigma, D1054) was added to stain reactive oxygen species (ROS) within the granulocytes. While reacting with ROS, DHR123 is oxidized to fluorescent rhodamine123 (Rho123). Samples were measured by flow cytometry (BD FACSCalibur) and analyzed with FlowJo™_V10 software.

### Flow Cytometry Analysis of *Streptococcus suis* Association With Porcine Monocytes and Lymphocytes

Peripheral blood mononuclear cells (PBMCs) were isolated from whole blood by a density gradient separation as described previously (Hohnstein et al., 2020). PBMCs (10^7^ cells/ml) were infected with S. suis*FR at an MOI of 1 for 30 min at 37°C, whereby *S. suis* had been pre-incubated in porcine serum of colostrum-deprived piglets (CDS) or in hyperimmune serum #4515 generated through prime-boost vaccination of a weaning piglet with a strain 10 bacterin (both obtained within previous studies; Weiße et al., 2021). Monocytes were stained using the myeloid marker CD172a-FITCs (BD Pharmingen™, 561498, 0.5 mg/ml). Lymphocytes were defined as CD172a-negative PBMCs. Samples were measured by flow cytometry (BD FACSCalibur) and analyzed with FlowJo™_V10 software. Following incubation with bacteria for 30 min or 2 h, in a subset of samples, PBMCs were stained with viability dye eFluor™ 506 (Thermo Fisher Scientific, 65-0866-14) according to manufacturer’s recommendations. These samples were measured using BD FACS Fortessa.

### Cytospin and Staining for Optical Microscopy

Directly after incubation of the PBMC samples with *S. suis*, an aliquot containing approximately 6 × 10^4^ PBMCs was added to 200 μl PBS and centrifuged onto slides for 5 min at 70 × *g* in a Shandon Cytospin 4. Slides were air dried and stained with a Diff-Quik staining kit (Medion Diagnostics, 726443) according to manufacturer’s recommendations. Microscopic analysis was performed using Olympus AX70 Provis microscope.

### Cytokine Quantification

DuoSet ELISA kits for porcine tumor necrosis factor (TNF)-α and IL-1β were purchased from R&D Systems (DY690B and DY681) and performed essentially according to manufacturer’s recommendations. The analysis was conducted with supernatants or plasma obtained before and after infection with *S. suis* in PBMC samples or bactericidal assays in whole blood as described above. The streptavidin-horseradish peroxidase used to couple the detection antibodies was detected with a 3,3′,5,5′-Tetramethylbenzidin (TMB) solution (SeraCare, Milford, MA, United States, formerly KPL) and the reaction was stopped after 20 min with 1 M H_3_PO_4_ (Roth, 6366.1). OD values were measured with a microplate reader SpectraMax 340PC384 (Molecular Devices, LLC San Jose, CA, United States) at 450 and 630 nm as a background reference and analyzed with SoftMax® Pro v5.0 software (Molecular Devices, LLC; Hohnstein et al., 2020).

### C3 Deposition on the Surface of *Streptococcus suis*

Deposition of complement on the streptococcal surface was measured essentially as described before (Rungelrath et al., 2018). Briefly, *S. suis* strains were grown to an OD_600_ of 0.5 in THB. Then, 50 μl of the respective culture was incubated with 100 μl of CDS for 1 h at 37°C under rotation (8 rpm). As negative control, CDS was incubated for 30 min at 56°C to inactivate all complement factors. Staining of C3 labeled bacteria was conducted with 200 μl of a 1:150 diluted FITC-labeled cross-reactive rabbit anti-human C3c antibody (Dako, F020102-2, 3 g/L) for 1 h at 4°C. Samples were measured using BD FACS Fortessa and analyzed using FlowJo™_V10 software.

### Anti-*Streptococcus suis* IgG and IgM ELISA

ELISAs measuring serum IgG or IgM binding to the surface of formaldehyde inactivated and immobilized *S. suis* strain 10 were conducted as described previously (Seele et al., 2015), using horseradish peroxidase (HRP) conjugated polyclonal goat anti-pig IgG (Bethyl, A100-105P) and goat anti-pig IgM (Bethyl, A100-117P) antisera. Hyperimmune serum #4515, which was also used in cell association assays, served as reference standard and was defined as containing 100 ELISA units of both IgG and IgM.

### Statistical Analysis

Statistical analysis was performed using Prism software, version 9 (GraphPad, San Diego, CA, United States). Normality was tested by Shapiro–Wilk test. Differences between multiple groups or time points were determined using ANOVA followed by Holm-Sídáks or Tukeys multiple comparisons test, respectively. Differences between wt and *dltA* mutant were tested using two-tailed paired *t*-test or Wilcoxon test when appropriate. Treatments with the two different porcine sera were compared by two-tailed unpaired *t*-test or Mann–Whitney test. A CI of 95% was chosen for all analyzes. All figures and data in parentheses in the text represent the means and SD. Probabilities were considered as indicated in the figure legends.

## Results

### Deletion of the *dltA* Gene Abolishes d-Alanylation of *Streptococcus suis* LTA

We created an isogenic mutant of the virulent serotype 2 strain 10 by in-frame deletion of the *dltA* gene, encoding for the d-alanine-d-alanyl carrier protein ligase in order to investigate the role of d-alanylation of LTAs in pathogen-host interaction. To verify the absence of alanine residues in the LTAs of *S. suis* 10Δ*dltA*, LTA was isolated from late exponential wt and mutant cultures and subjected to NMR spectroscopy. Overall, the ^1^H NMR spectrum recorded for LTA isolated from the wt (Supplementary Figure S1, top panel) is virtually identical with those obtained earlier for LTA isolated from other *S. suis* serotype 2 strains (strains P1/7 and SC84; Gisch et al., 2018) and further NMR analysis confirmed the identical overall structure. The peaks indicative for d-alanine residues present at the position O-2 of glycerol within the poly-(glyco)glycerolphosphate chain [δ_H_ 1.65–1.60 (Ala-CH_3_), 4.33–4.27 (Ala-CH), and 5.42–5.36 (glycerol-H2) ppm; Fittipaldi et al., 2008; Gisch et al., 2018] were absent in the spectra recorded from LTA of the Δ*dltA* strain (Supplementary Figure S1). This result confirms that the *dltA* gene is necessary for d-alanylation of LTA in *S. suis*, as already described previously (Fittipaldi et al., 2008).

### mRNA Expression Analysis of the dlt Operon and Adjacent Genes

Quantitative real-time PCR verified the absence of mRNA expression of *dltA* in the mutants 10Δ*dltA* and 10cpsΔEFΔ*dltA* (Supplementary Figure S2). Transcript levels of the genes *dltC* and *dltD*, as well as those of the genes SSU10_RS03040 and SSU10_RS03090 up- and downstream of the dlt locus, were not different between *S. suis* 10 wt and 10Δ*dltA*, confirming that in-frame deletion did not result in polar effects. The non-encapsulated mutant 10cpsΔEF showed a slightly increased ΔΔCT of 1.8 (SD 0.36) for SSU10_RS03040 in comparison with *S. suis* strain 10 wt, whereas the double mutant 10cpsΔEFΔ*dltA* obtained a slightly decreased ΔΔCT of 0.5 (SD 0.22) for SSU10_RS03040. This open reading frame encodes putatively a glucosamine-6-phosphate deaminase, which is called NagB in *Staphylococcus aureus* and *Streptococcus mutants* (Komatsuzawa et al., 2004; Kawada-Matsuo et al., 2016). NagB is responsible for the synthesis of fructose from glucosamine. High concentrations of amino sugars like N-acetylglucosamine (GlcNAc) in the medium result in upregulation of the expression of NagB (Kawada-Matsuo et al., 2016). We speculate that the slightly modified expression of SSU10_RS03040 in the non-encapsulated mutants might be related to the deletion of the capsule biosynthesis genes, as GlcNAc is part the capsular polysaccharide of *S. suis* serotype 2 (van Calsteren et al., 2010).

### Effects of LTA d-Alanylation on Surface Charge, Hydrophobicity, and Susceptibility to Cationic Antimicrobial Peptides

**Figure 1:**
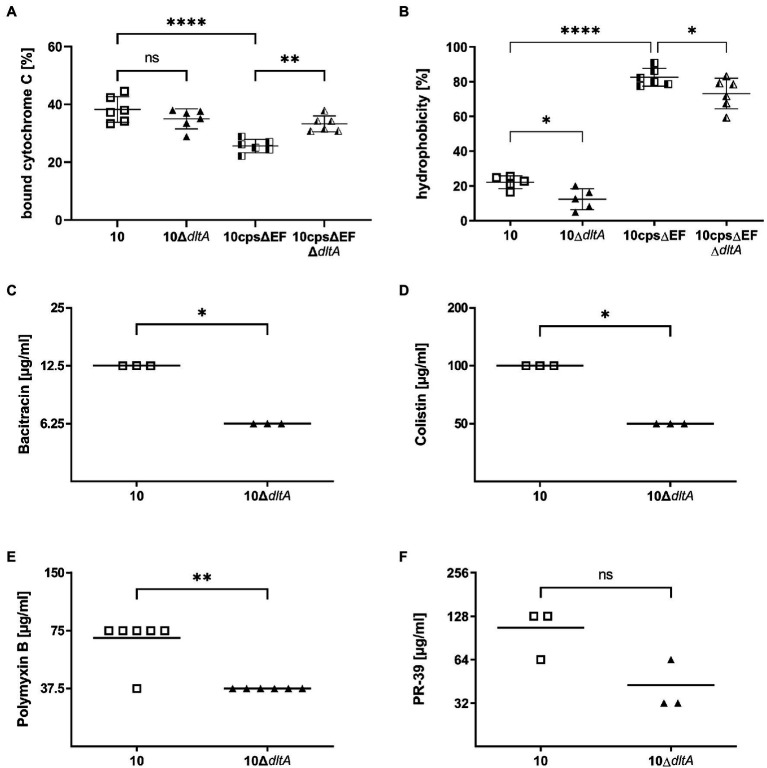
D-alanylation of lipoteichoic acids (LTAs) increases the ability to bind cationic cytochrome C in the absence of the capsule in *Streptococcus suis*, reduces the hydrophobic character of the streptococcal surface, and provides protection against several cationic antimicrobial peptides (CAMPs). Binding of the cationic cytochrome C (Cyt C) to the bacterial surface **(A)** was evaluated indirectly by photometrical measurement of the bacterial supernatants after 10 min of incubation with 0.5 mg/ml Cyt C in morpholinepropanesulfonic acid (MOPS) buffer, compared to a control containing 0.5 mg/ml Cyt C without bacteria. **(B)** Shows the hydrophobicity of *S. suis* strains, as evaluated by rigorous mixing of bacterial PBS suspensions (adjusted to OD_600_ of 1) with the hydrophobic solvent hexadecane. The mixtures were left to settle for 30 min followed by measuring the OD_600_ of the aqueous (PBS) phase and calculation of the hydrophobicity as written in the materials and methods section. **(C–F)** Demonstrate minimal bactericidal concentrations (MBC) that were evaluated by incubating *S. suis* strain 10 or 10Δ*dltA* (1 × 10^4^ CFU/ml) in a 2-fold serial dilution of the antimicrobial agent for 24 h at 37°C and subsequently plating on blood agar plates. MBC was defined as the lowest concentration of the antimicrobial agent where no bacterial growth occurred on agar plates. Each symbol represents a measurement in triplicates and each experiment was repeated at least three times with freshly cultured bacteria. Assays were performed in Todd-Hewitt broth (THB; **C,D,E**) or Rosewell Park Memorial Institute (RPMI) with 5% cation-adjusted Mueller Hinton broth (CA-MHB; **F**). One-way ANOVA was performed followed by Holm-Sídáks multiple comparisons test and results are indicated for the comparisons of each isogenic mutant to its parent strain **(A,B)**. One-tailed Mann–Whitney test was performed to compare wild-type (wt) and *dltA* mutant samples in **(C–F)**. Not significant (ns) *p* ≥ 0.05, ^*^p < 0.05, ^**^p < 0.01, and ^****^p < 0.0001.

We hypothesized in agreement with results for Group A streptococci (Kristian et al., 2005) and pneumococci (Saar-Dover et al., 2012) that the net negative charge of the bacteria is increased in the two Δ*dltA* mutants. However, we found no significant difference in binding of the cationic peptide cytochrome C between *S. suis* wt and 10Δ*dltA* (Figure 1A; strain 10: mean 38.27%, SD 4.43%; 10Δ*dltA*: mean 34.99%, SD 3.46%). In contrast, loss of LTA d-alanylation in the non-encapsulated mutant caused a highly significant increase in bound cytochrome C (10cpsΔEF: mean 25.57%, SD 2.32%; 10cpsΔEFΔ*dltA*: mean 33.25%, SD 2.76%). These results suggest that changes in local charges due to LTA d-alanylation in the wt bacteria are masked by the capsular polysaccharides. We further investigated hydrophobicity of the different streptococci by the microbial adhesion to hydrocarbons assay. While the capsule provided a hydrophilic character to the streptococcal surface and its absence caused a strongly significant increase of hydrophobicity (strain 10: mean 22.18%, SD 3.66%; 10cpsΔEF: mean 82.60%, SD 5.10%), 10Δ*dltA* and 10cpsΔEFΔ*dltA* showed a significantly reduced cell surface hydrophobicity compared to the wt and the capsular mutant 10cpsΔEF, respectively (10Δ*dltA*: mean 12.39%, SD 6.08%; 10cpsΔEFΔ*dltA*: mean 73.24%, SD 8.80%; Figure 1B). In conclusion, LTA d-alanylation increases cell surface hydrophobicity of encapsulated as well as non-encapsulated *S. suis*.

We also determined the MBC of the bacteria-derived peptides bacitracin, colistin, and polymyxin B and the porcine cathelicidin PR-39 (Figures 1C–F). Bacitracin, colistin, and polymyxin B showed a significantly lower MBC for 10Δ*dltA* than for the wt strain, confirming and expanding the results obtained for MIC of an independent laboratory (Fittipaldi et al., 2008). Thus, it has been shown by independent laboratories that d-alanylation in *S. suis* serotype 2 decreases the susceptibility to several CAMPs.

### The Oxidative Burst Response of Porcine Granulocytes and the Association With *Streptococcus suis* Is Reduced by d-Alanylation of LTAs

**Figure 2:**
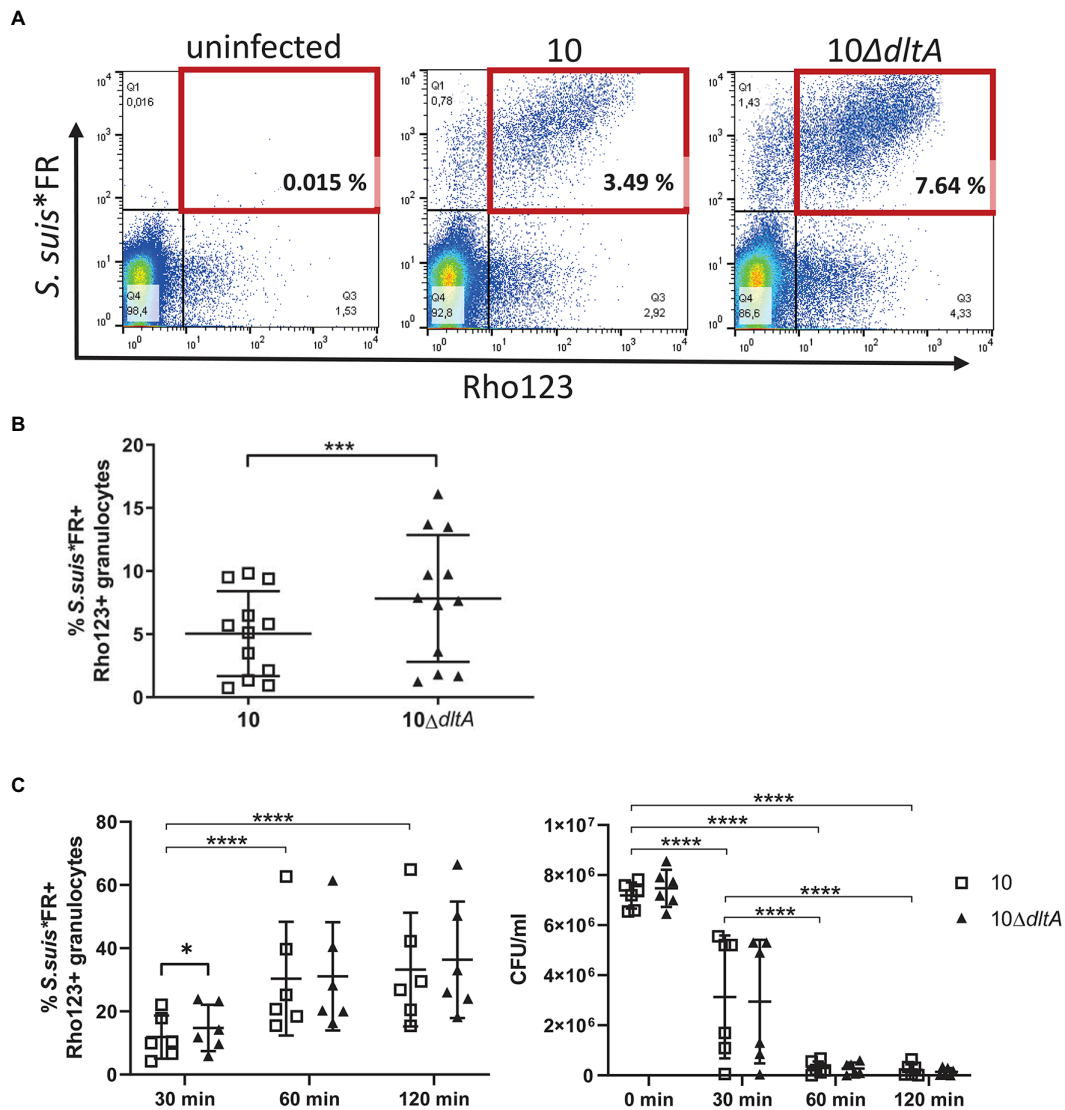
D-alanylation of LTAs in *Streptococcus suis* reduces early association of *S. suis* with granulocytes and the proportion of granulocytes performing oxidative burst. Whole porcine blood was infected with Far Red (FR)-labeled *S. suis* at a concentration of 1 × 10^7^ CFU/ml at 37°C for 15 min, followed by intracellular staining of reactive oxygen species (ROS) with dihydrorhodamine123 (Rho123) at 37°C for 10 min. After erythrocyte lysis, samples were analyzed by flow cytometry. While gating of granulocytes in whole blood is depicted in Supplementary Figure S4A, **(A)** demonstrates the gating strategy for double positive granulocytes (red square) in the blood of one piglet and **(B)** shows the % of granulocytes interacting with S. suis*FR (FR+) and producing ROS at the same time (Rho123+; *n* = 12). **(C)** Demonstrates the course of the association of streptococci with granulocytes that simultaneously show oxidative burst response over time, as well as the CFU of S. suis*FR in blood of six piglets. Error bars represent SDs. Paired *t*-test was performed for statistical analysis to compare wt and mutant samples in blood of the same animals **(B,C)** and repeated measures ANOVA followed by Tukeys multiple comparisons test was performed for each strain at different time points **(C)**. For clarity, significant differences between the time points are indicated for the wt samples only but are equally seen for the *dltA* mutant. ^**^p < 0.01, ^***^p < 0.001, and ^****^p < 0.0001.

The role of LTA d-alanylation in protection of streptococci against neutrophils is described in different studies (Poyart et al., 2003; Kristian et al., 2005; Fittipaldi et al., 2008). To specifically investigate whether d-alanylation of LTAs influences phagocytosis of *S. suis* by porcine granulocytes during bacteremia, whole blood of 8-week-old piglets was infected with Far Red (FR)-labeled bacteria (S. suis*FR). The labeling of bacteria was verified by flow cytometry (Supplementary Figure S3). Flow cytometry analysis of the infected blood samples enabled us to read out the number of granulocytes associated with S. suis*FR but did not allow us to distinguish extracellular binding or intracellular presence of *S. suis* within the granulocytes. Therefore, measurement of *S. suis*-granulocytes association was combined with the evaluation of ROS production to identify granulocytes performing phagocytosis. Gating of double positive granulocytes is visualized exemplarily for one blood donor in Figure 2A.

We found a higher number of granulocytes in association with strain 10Δ*dltA* (FR positive granulocytes) generating ROS (Rho123 positive) than observed for the wt (10Δ*dltA*: mean 7.83% and SD 5.03%; strain 10: mean 5.04% and SD 3.34%), indicating a higher level of phagocytosis of the Δ*dltA* mutant (Figure 2B). The observation that d-alanylation might protect *S. suis* from phagocytosis and oxidative burst by granulocytes goes in line with the described increased killing of the *S. suis dltA* mutant by neutrophils (Fittipaldi et al., 2008).

To answer the question, whether increased oxidative burst and association of granulocytes with the *dltA* mutant result in an increased killing of the mutant in whole blood, we investigated the oxidative burst and streptococci-cell association at different time points and plated the Far Red-labeled bacteria at the same time points (Figure 2C). Again, we saw a higher percentage of granulocytes in association with the 10Δ*dltA* mutant, while performing oxidative burst after 30 min of incubation (10Δ*dltA*: mean 14.78%, SD 7.32%; strain 10: mean 11.88% SD 6.81%), while after 1 and 2 h differences between wt and *dltA* mutant were not significant. During time, the mean percentage of granulocytes showing oxidative burst simultaneously to association with *S. suis* significantly increased for both strains and reached values around 33 and 36% after 2 h. In all six blood donors, the bacteria were efficiently killed with significant reduction of the mean CFU already after 30 min. These results suggest that d-alanylation of LTA reduces the early association with granulocytes and their activation as *S. suis* enters porcine blood but might not ensure efficient escape in the progress of infection, at least not in the blood of conventional 8-week-old piglets that generally carry specific IgG and IgM antibodies against *S. suis* but are nevertheless often affected by *S. suis* disease.

### LTA d-Alanylation Reduces the Level of Association of *Streptococcus suis* With Porcine Monocytes

The interaction of *S. suis* with porcine brain microvascular endothelial cells can be inhibited through addition of LTA (Vanier et al., 2007). Furthermore, d-alanylation of LTA is described to influence the interaction of the streptococci with dendritic cells (Lecours et al., 2011). We hypothesized that LTA and its modification through d-alanylation might also modulate the interaction with porcine monocytes in blood.

**Figure 3:**
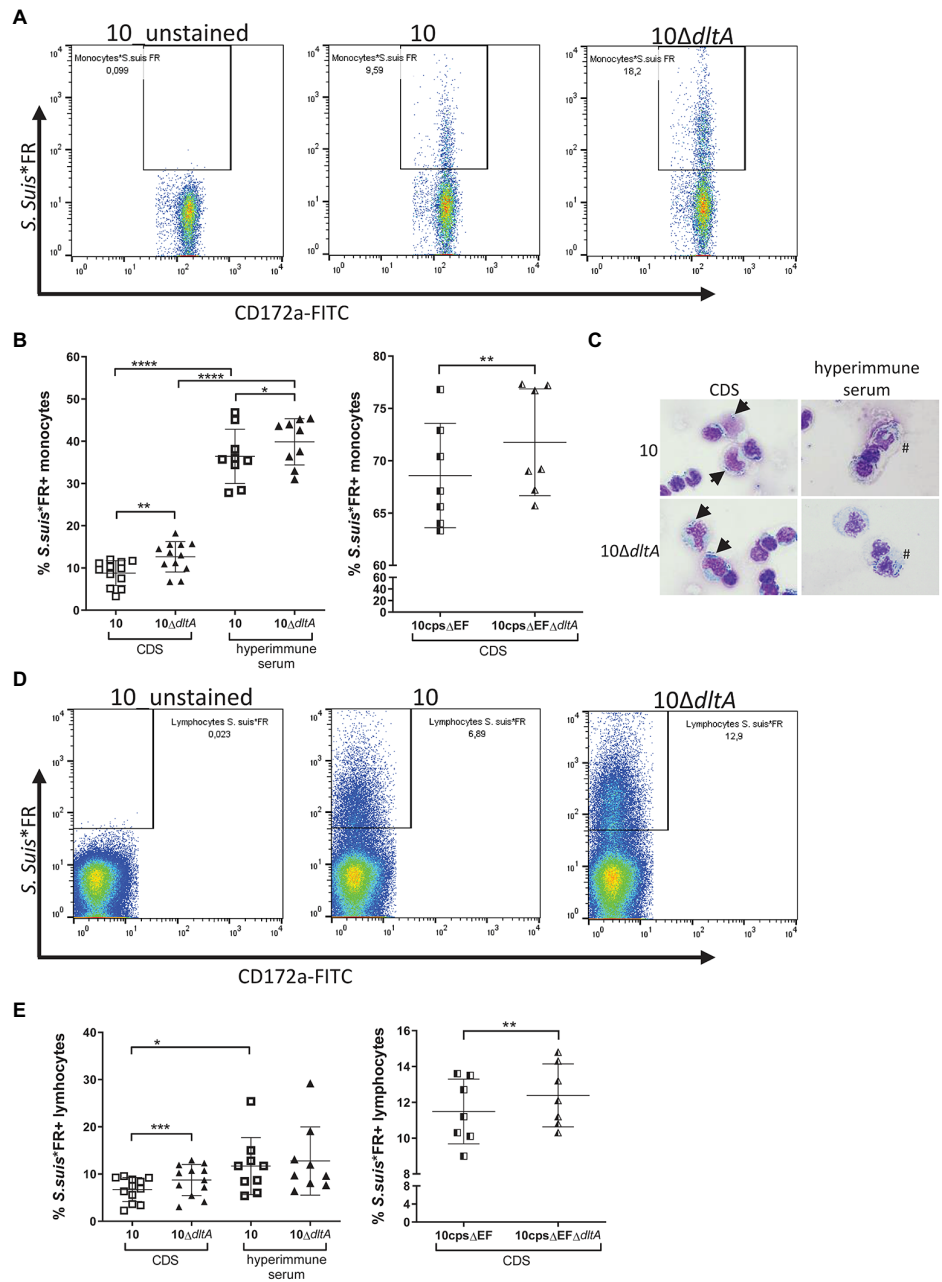
Far Red-labeled *Streptococcus suis* Δ*dltA* mutants show a significantly higher level of association with porcine monocytes and lymphocytes. Peripheral blood mononuclear cell (PBMCs; freshly isolated from porcine blood) were incubated with Far Red-labeled *S. suis* strains (S. suis*FR) at an MOI of 1 for 30 min at 37°C, whereby S. suis*FR had been pre-incubated in serum of colostrum-deprived piglets (CDS) or in hyperimmune serum for 30 min, as indicated. Monocytes were stained using the myeloid marker CD172a-FITCs (as visualized in Supplementary Figure S4) and samples were measured by flow cytometry. The gating strategy for monocytes **(A)** and lymphocytes **(D)** in association with *S. suis* is visualized for PBMCs of one animal, while (**B**; monocytes) and (**E**; lymphocytes) show the entire results for the encapsulated strains on the left and the non-encapsulated strains on the right. The number of symbols represents the number of piglets PBMCs were obtained from. **(C)** Shows exemplary pictures of CDS-treated *S. suis* bound to the surface of monocytes (arrow) and uptake of antibody-opsonized *S. suis* (#) into monocytes as visualized by optical microscopy (x400 magnification). Horizontal lines and error bars represent mean values and SDs. For statistical analysis, paired *t*-test (wt or 10cpsΔEF vs. respective Δ*dltA* mutant) or unpaired *t*-test (CDS vs. hyperimmune samples) were performed. In case of not normally distributed samples, Wilcoxon test or Mann–Whitney test was used. Differences that are not indicated are not significant. ^*^p < 0.05, ^**^p < 0.01, ^***^p < 0.001, and ^****^p < 0.0001.

Accordingly, we isolated PBMCs from freshly obtained porcine blood and applied S. suis*FR strain 10 or 10Δ*dltA* at an MOI of 1. The myeloid marker CD172a was used to identify monocytes (Supplementary Figure S4B) and samples were analyzed for FR positive monocytes by flow cytometry (Figure 3A). To evaluate the impact of serum components on the bacteria-cell-association, we pre-incubated the streptococci with either serum of CDS, containing no specific antibodies against *S. suis*, or with hyperimmune serum raised against *S. suis* strain 10.

In the absence of specific antibodies (CDS-treated bacteria), LTA d-alanylation significantly reduced the level of association of *S. suis* with monocytes (Figure 3B, left graph, CDS; strain 10: mean 8.78%, SD 2.95%; 10Δ*dltA*: mean 12.63%, SD 3.6%), while the association was strongly enhanced for both strains when bacteria were opsonized with specific antibodies (Figure 3B, left graph, hyperimmune serum; strain 10: mean 36.41%, SD 6.43%; 10Δ*dltA*: mean 39.87%, SD 5.48%). In these hyperimmune conditions, the influence of LTA d-alanylation on the association of *S. suis* with monocytes remained significant but less pronounced. In microscopic analysis, the association of monocytes with CDS-treated *S. suis* resembled a binding of the streptococci to the cell surface, whereas opsonization with hyperimmune serum partly resulted in streptococci visible within the membrane borders of monocytes, indicating a possible uptake by phagocytosis (Figure 3C).

As the capsule of *S. suis* strongly influences its interaction with mononuclear cells (Smith et al., 1999; Meijerink et al., 2012), we repeated the assays using the non-encapsulated mutant 10cpsΔEF and the double mutant 10cpsΔEFΔ*dltA* to exclude the influence of capsular polysaccharides on the *dltA* phenotype. Even in the absence of specific antibodies, the non-encapsulated mutants showed a very high level of association with monocytes (Figure 3B, right graph, 10cpsΔEF: mean 68.59%, SD 4.99%) and the loss of LTA d-alanylation significantly increased the number of FR positive monocytes (10cpsΔEFΔ*dltA*: mean 71.76%, SD 5.10%), similar to what was observed for the encapsulated strains. These results indicate that d-alanylation of LTA modulates association of *S. suis* with monocytes independently of the capsular polysaccharides.

When analyzing the association of the streptococci with lymphocytes contained in the PBMC samples (Figure 3D), we found that the bacteria also adhered to lymphocytes in a *dltA*-dependent manner when no specific antibodies where present, as CDS-treated 10Δ*dltA* showed a significantly increased association with lymphocytes (Figure 3E, left graph, CDS; strain 10: mean 6.73%, SD 2.53%; 10Δ*dltA*: mean 8.74%, SD 3.28%). The same was visible in the non-encapsulated strains, although the increase was small (Figure 3E, right graph; 10cpsΔEF: mean 11.48%, SD 1.81%; 10cpsΔEFΔ*dltA*: mean 12.39%, SD 1.76%). In contrast to monocytes, the increase of lymphocytes associated with *S. suis* due to opsonization with specific antibodies was far less pronounced (Figure 3E, left graph, hyperimmune serum; strain 10: mean 11.68%, SD 6.04%; 10Δ*dltA*: mean 12.76%, SD 7.24%) and an influence of LTA d-alanylation was no longer detected.

**Figure 4:**
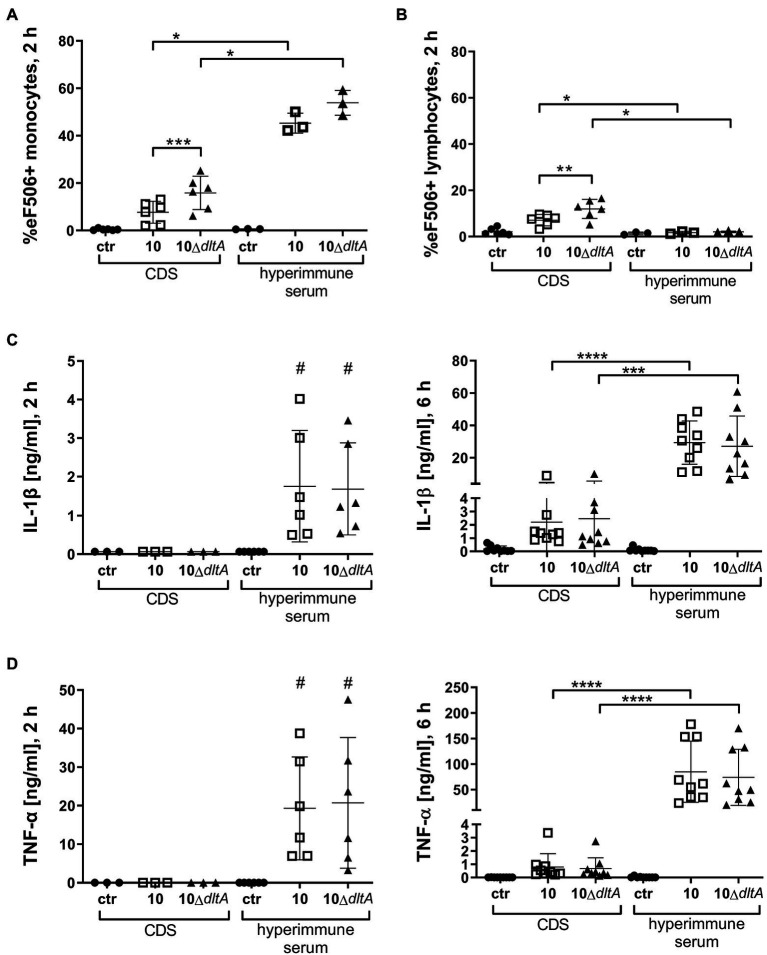
Infection of PBMCs with *Streptococcus suis* opsonized in hyperimmune serum induces membrane damage in monocytes but not in lymphocytes and results in induction of IL-1β and tumor necrosis factor (TNF)-α. For **(A,B)**, a subset of PBMC samples visualized in Figure 3 (*n* = 6 for CDS and *n* = 3 for hyperimmune serum) were stained with the viability dye eFlour™ 506 after 2 h of incubation with the respective *S. suis* strains. PBMC samples without bacteria served as negative control while heat-treated PBMCs served as a positive control (not shown: eF506+ monocytes 90.9%; eF506+ lymphocytes 89.6%). Samples were measured by flow cytometry. Results for monocytes are visualized in **(A)**, while lymphocytes are depicted in **(B)**. Pro-inflammatory cytokines IL-1β **(C)** and TNF-α **(D)** were measured by ELISA in the supernatant of PBMC samples visualized in Figure 3 after 2 and 6 h of infection with respective *S. suis* strains. The limit of detection was 0.031 ng/ml for TNF-α and 0.063 ng/ml for IL-1β. Horizontal lines and error bars represent mean values and SDs. For statistical analysis, paired *t*-test (wt vs. mutant) or unpaired *t*-test (CDS vs. hyperimmune samples) was performed. In case of not normally distributed samples **(C,D)**, Wilcoxon test or Mann–Whitney test was used, respectively. One sample Wilcoxon was performed to evaluate how much IL-1β and TNF-α levels differ from the values 0.063 or 0.031 ng/ml, respectively, after 2 h of incubating PBMCs with *S. suis* opsonized in hyperimmune serum (all other values in this setting lay below the detection limit; #). ^*/#^*p* < 0.05, ^**^p < 0.01, ^***^p < 0.001, and ^****^p < 0.0001.

To verify our cell association data and exclude falsification of data due to cell death, staining with Viability Dye eFluor™ 506 (eF506) was performed with the PBMC samples and the percentage of cells containing eF506 was measured after 30 min (Supplementary Figure S5) and after 2 h of incubation with *S. suis* (Figure 4A). After 30 min, which corresponds to the time of evaluation of the bacterial association with monocytes, the number of cells displaying membrane damage (positive for eF506), in samples incubated with CDS-treated bacteria, was still very low and comparable to samples without bacteria (Supplementary Figure S5). In contrast, after 2 h of incubation, CDS-treated 10Δ*dltA* induced a higher number of eF506 positive monocytes than CDS-treated wt strain 10 (Figure 4A CDS; strain 10: mean 7.70%, SD 4.58%; 10Δ*dltA*: mean 15.85%, SD 7.02%). When bacteria were opsonized with specific antibodies contained in the hyperimmune serum, we found very high percentages of eF506 positive monocytes 2 h after infection with the wt (mean 45.30%, SD 4.21%) or the Δ*dltA* mutant (mean 53.87%, SD 5.25%; Figure 4A hyperimmune serum). The percentage of eF506 positive lymphocytes did not increase upon incubation with the opsonized *S. suis* (Figure 4B hyperimmune serum; 10 mean 1.73%, SD 0.50%; 10Δ*dltA* mean 2.03%, SD 0.36%) but was reduced compared to the samples incubated with CDS-treated bacteria (Figure 4B CDS; 10 mean 7.11%, SD 2.45%; 10Δ*dltA* mean 11.95%, SD 4.13%).

As we were interested in the immunomodulatory effect of the association of *S. suis* with monocytes, we measured the pro-inflammatory cytokines IL-1β and TNF-α in the supernatants of the infected PBMC samples after 2 and 6 h. Only in supernatants of PBMCs infected with bacteria pre-treated with hyperimmune serum, we found both cytokines already after 2 h of infection (Figures 4C,D). In samples with CDS-treated *S. suis*, the cytokines were undetectable at 2 h and still quite low at 6 h post-infection. There was no significant difference in the levels of the two cytokines between samples infected with *S. suis* wt or 10Δ*dltA*, but when *S. suis* was pre-opsonized in hyperimmune serum, it induced significantly higher levels of both pro-inflammatory cytokines, than without opsonization with specific antibodies (Figures 4C,D).

In conclusion, d-alanylation of LTA significantly reduces association of *S. suis* with porcine monocytes in isolated PBMCs infected *ex vivo*. Independent of LTA d-alanylation, opsonization with specific antibodies results in a strong increase of this association, followed by membrane damage in monocytes and induction of the pro-inflammatory cytokines IL-1β and TNF-α.

### In the Absence of Specific Antibodies Complement Deposition on the Surface of *Streptococcus suis* Is Reduced Through d-Alanylation of LTAs

**Figure 5:**
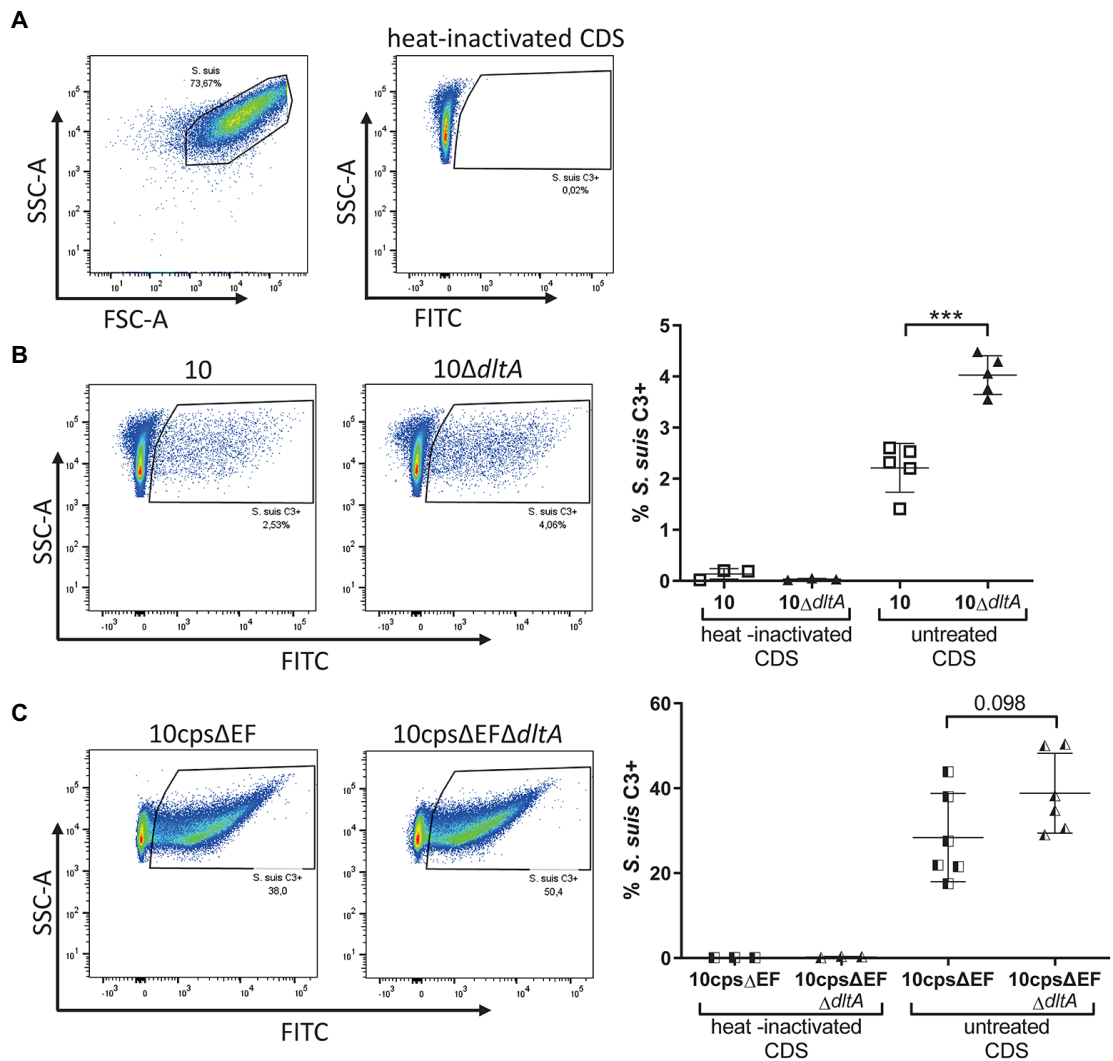
Labeling with complement factor C3 is increased in *Streptococcus suis* 10Δ*dltA* after incubation in active CDS. *Streptococcus suis* strains were grown to early exponential phase (OD_600_ of 0.5) in THB and incubated with CDS for 1 h, followed by staining of C3 with a cross-reactive FITCs conjugated anti-human C3c antibody and measurement of C3 associated with the bacterial surface by flow cytometry. **(A)** Represents the gating strategy and negative control sample, while (**B**; encapsulated *S. suis*) and (**C**; unencapsulated *S. suis*) visualize representative examples on the left and the complete data sets on the right. Horizontal lines and error bars represent mean values and SDs. For statistical analysis, unpaired *t*-test was used to compare parent strain and respective Δ*dltA* mutant. ^***^p < 0.001.

As suggested previously (Lecours et al., 2011), LTA d-alanylation might reduce complement deposition on the bacterial surface resulting in reduced interaction with myeloid cells such as dendritic cells. Since, we saw LTA d-alanylation-dependent differences in the association of *S. suis* with monocytes, we asked if the level of C3 deposition on the surface of CDS-treated *S. suis* depends on d-alanylation of LTAs. Flow cytometry was used to investigate opsonization of streptococci with C3 (Figure 5A). A significantly increased number of 10Δ*dltA* bacteria were stained positive for C3 on their surface compared to the wt (Figure 5B, strain 10 mean 2.21%, SD 0.48%; 10Δ*dltA* mean 4.03%, SD 0.38%). A similar tendency was visible for non-encapsulated *S. suis*, whereby the numbers of bacteria opsonized with C3 were higher than observed for the encapsulated strains (Figure 5C, 10cpsΔEF: mean 28.38%, SD 10.41%; 10cpsΔEFΔ*dltA*: mean 38.82%, SD 9.40%). For all strains, C3 deposition on bacteria incubated with heat-inactivated CDS was nearly absent, confirming that the measurement of C3 deposition depends on active complement. In conclusion, our results indicate that LTA d-alanylation is involved in complement evasion.

### Mutation of the *dltA* Gene of *Streptococcus suis* Reduces the Amount of IL-1β but Not of TNF-α Induced by *Streptococcus suis* in Porcine Blood

**Figure 6:**
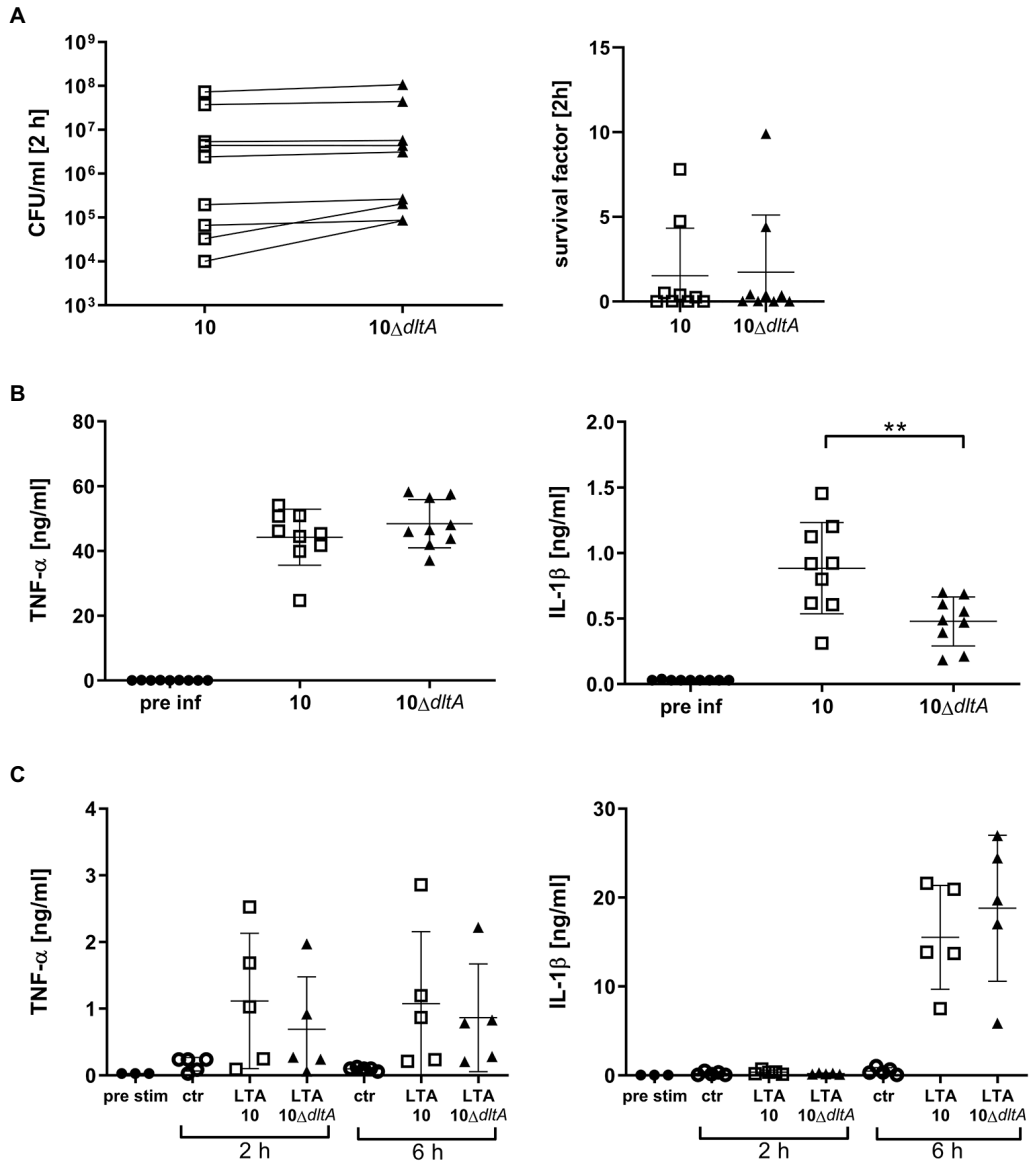
*Streptococcus suis* strain 10 wt induces comparable levels of TNF-α, but higher levels of IL-1β in porcine blood than the mutant 10Δ*dltA*, while purified LTAs of wt and 10Δ*dltA* do not show differences in IL-1β induction upon addition to porcine blood. Whole porcine blood was infected with *S. suis* strain 10 and 10Δ*dltA* at an inital CFU of 1 × 10^7^/ml. After 2 h infection, CFUs and survival factor **(A)** as well as TNF-α and IL-1β levels in plasma **(B)** were determined. For **(C)**, blood was treated with 30 μg/ml purified LTA obtained from strain 10 or 10Δ*dltA* as indicated, whereby citric acid was used as a solvent for LTA. The equivalent volume of citric acid was used in control samples (ctr). Plasma was obtained after incubation as well as prior to infection (pre-inf) or stimulation (pre-stim), respectively, to determine cytokine levels by ELISA. The limit of detection was 0.016 ng/ml for TNF-α and 0.032 ng/ml for IL-1β and all pre-inf and pre-stim samples lay below that limit. Lines in the left graph in **(A)** connect results of each individual blood donor. Horizontal lines and error bars represent mean values and SDs, respectively. For statistical analysis, paired *t*-test **(A,B)** or Wilcoxon matched-pairs signed rank test **(C)** was performed comparing wt and mutant samples. Differences that are not indicated are not significant. ^**^p < 0.01.

To investigate the pro-inflammatory response in *S. suis* infected porcine blood in dependence of d-alanylation of LTAs, we focused on two differently regulated pro-inflammatory cytokines, namely, IL-1β and TNF-α. Again, the lack of LTA d-alanylation made no difference in bacterial survival after 2 h of infection observed in the blood of these animals originating from the same conventional herd as shown in Figure 2 (Figure 6A, SF strain 10: mean 1.52, SD 2.80; 10Δ*dltA* mean 1.73, SD 3.38). However, while the number of CFU after 2 h in porcine blood was comparable between wt and the isogenic Δ*dltA* mutant, a distinct pattern of cytokine production was recorded. Whereas the amount of TNF-induced by the two strains was very similar, the wt induced with 0.88 ng/ml (SD 0.35) significantly higher amounts of IL-1ß than the *dltA* mutant with 0.48 ng/ml (SD 0.18; Figure 6B). This phenotypical difference, which we had not observed in isolated PBMCs, was reproducible in blood obtained from animals of an independent herd with a different history of *S. suis* diseases, mainly dominated by serotype 9 (Supplementary Figure S6).

Purified LTAs obtained from strain 10 and 10Δ*dltA* were applied to porcine blood (final LTA concentration 30 μg/ml), which was subsequently incubated for 2 and 6 h. The concentrations of TNF-α and IL-1β after 2 and 6 h varied between samples but did not seem to depend on the *dltA* genotype of the original strain. Of note, IL-1β levels were below the limit of detection of 0.032 ng/ml after 2 h but above 5 ng/ml (mean 15.53 and 18.80 ng/ml for LTAs of wt and 10Δ*dltA*, respectively) after 6 h indicating a delayed induction of IL-1β secretion by the purified LTAs in comparison with whole living *S. suis* in this assay (Figure 6C).

In conclusion, infection of porcine blood *ex vivo* with *S. suis* wt and 10Δ*dltA* indicates that d-alanylation of LTAs results in increased secretion of IL-1β, while the level of alanylation of purified LTAs, used for *ex vivo* stimulation of porcine blood, did not influence the amount of secreted IL-1β. Therefore, the reduction of IL-1β levels, observed in blood infected with the Δ*dltA* mutant, is most likely not attributable to a directly reduced activation of PRRs in porcine immune cells by the LTAs of 10Δ*dltA* compared to the LTAs of the wt.

## Discussion

More than a decade ago, Fittipaldi et al. (2008) described d-alanylation of LTA in *S. suis* as a virulence mechanism. We revisited this phenotype and confirmed important findings of this previous study by independent loss-of-function experiments, namely, that *dltA* is necessary for d-alanylation of LTA and that an isogenic Δ*dltA* mutant is more susceptible to the antimicrobial agents colistin and polymyxin B.

When determining MBCs, we found an increased susceptibility of 10Δ*dltA* to several CAMPs, and as a tendency also for the cathelicidin PR-39, which is found in porcine NET structures (de Buhr et al., 2017). A common explanation for the protective effect of LTA d-alanylation is the introduction of positive charges into the bacterial cell wall, increasing the net positive charge of the bacterial surface and resulting in an increased electrostatic repulsion of CAMPs. In contrast to this theory, we did not find an increase of the binding of cationic cytochrome C to the encapsulated Δ*dltA* mutant, suggesting no difference to the wt in the net surface charge. A similar inconsistency was described by Poyart et al. (2003), when transmission electron microscopy of *Streptococcus agalactiae* showed stronger binding of positively charged metal ions to the wt rather than to the *dltA* mutant. It was discussed that the resulting local charge variation causes structural modifications that render the metal binding sites less accessible in the wall of the *dltA* mutant. However, for the observed increase in resistance to CAMPs, it might not be crucial for *S. suis* to increase its net surface charge. Saar-Dover et al. (2012) suggest that the anionicity of the bacterial surface does not modulate the accumulation of CAMPs and the increased resistance resulting from d-alanylation of Group B *Streptococcus* LTA might rather be attributed to an altered conformation of the LTAs and resulting modulations in the density and surface properties of the bacterial cell wall. Therefore, even without increasing the global charge, LTA d-alanylation could contribute to CAMP resistance in *S. suis*. In the absence of the capsule, we observed an effect of LTA d-alanylation on the electric charge of the bacterial surface as determined by cytochrome C binding. The non-encapsulated Δ*dltA* mutant bound a significantly higher amount of cytochrome C than its non-encapsulated parent strain. This suggests that d-alanylation of LTA makes a difference for the electric charge of the bacterial surface if the capsule is downregulated and that capsular polysaccharides of *S. suis* can mask this electric charge. Furthermore, we found an increased surface hydrophobicity due to the introduction of d-alanine into LTAs of *S. suis* as determined by the microbial adhesion to hydrocarbons assay. This might be caused by the hydrophobic character of the methyl group in alanine.

Many Gram-positive bacteria do not only possess LTA but also teichoic acids covalently attached to peptidoglycan, the so-called wall teichoic acids (WTAs; Brown et al., 2013). Deletion of the *dltA* gene has been shown to abolish d-alanylation in both TA polymers (Perego et al., 1995; Peschel et al., 1999). This might also be the case for *S. suis*. Although, the presence of WTAs in *S. suis* has not yet been proven on the molecular level, genes encoding members of the LCP family (LytR-CpsA-Psr), shown to be involved in cell wall anchoring of WTAs in *Staphylococcus aureus* (Chan et al., 2013; Schaefer et al., 2017) or *Bacillus subtilis* (Gale et al., 2017), namely, LytR and Psr, have also been identified in *S. suis* (Huang et al., 2021). Therefore, it seems likely that the phenotypes observed due to *dltA* deletion in *S. suis* are also partly attributable to a resulting loss of WTA d-alanylation.

Neutrophils represent a large part of the immune cells in blood and are major players in controlling bacteremia during *S. suis* infection. Comparing *S. suis* Δ*dltA* to its wt serotype 2 strain, Fittipaldi et al. (2008) describe increased killing of the mutant by porcine neutrophils, although the performed neutrophil killing assay was not able to distinguish between intra- and extracellular killing. Flow cytometry analysis enabled us to further clarify the role of LTA d-alanylation in the interaction of *S. suis* with neutrophils. After 30 min of infection, we observed a larger number of granulocytes in association with 10Δ*dltA* and simultaneously presenting an oxidative burst response in whole porcine blood, indicating an increased phagocytosis of the Δ*dltA* mutant compared to the wt. This is also in accordance with findings of Fittipaldi et al. (2008) showing increased killing of the Δ*dltA* mutant by porcine neutrophils after opsonization with complete porcine serum. Although antibody- and complement-dependent phagocytosis and oxidative burst of granulocytes are important to limit the survival of *S. suis* (Rungelrath et al., 2020), we did not record reduced killing of the Δ*dltA* mutant in whole porcine blood. In contrast, the specific bacterial content in the blood of a specific piglet was very similar between the wt and the isogenic mutant (Figure 6A), though there were substantial differences between piglets and blood of most piglets demonstrated killing of wt and mutant. Monitoring bacterial survival, *S. suis*-granulocyte association and oxidative burst over 2 h in whole blood of six piglets, proofed an efficient killing of both *S. suis* strains and, in parallel, a significant increase of association and oxidative burst. This is most likely due to opsonophagocytosis, with antibodies masking the initially observed effect of LTA d-alanylation on phagocytosis. We investigated bacterial survival in blood of 8-week-old conventional piglets which carried IgG and IgM antibodies binding to the surface of *S. suis* strain 10 as shown by ELISA (Supplementary Figure S7). Of note, this age class is severely affected by *S. suis* diseases in the field though IgG and IgM antibodies binding to streptococcal surface antigens are generally detectable in these piglets (Rieckmann et al., 2018; Mayer et al., 2021). We did not carry out bactericidal assays in the absence of specific antibodies, but based on results of previous investigations (unpublished results and Rungelrath et al., 2020), we speculate that wt and its isogenic *dltA* mutant will both exhibit high proliferation rates under such conditions.

The analysis of PBMC samples showed that d-alanylation of LTAs also reduces the association of *S. suis* with monocytes and lymphocytes. Macrophages/monocytes, polymorphonuclear leukocytes, B-lymphocytes, and subpopulations of T-lymphocytes have been shown to express the complement receptor 1 which binds to complement protein C3b on the bacterial surface (Vandendriessche et al., 2021). As already suggested by Lecours et al. (2011) when working with *S. suis* and dendritic cells of mice, a reduction in complement opsonization could be the underlying protective mechanism caused by the d-alanylation of LTA. Accordingly, the Δ*dltA* mutant showed an increased level of C3 deposition on its surface after incubation in serum that does not contain specific antibodies against *S. suis*. We assume that the influence of d-alanylation of LTAs on complement activation is mainly put down to the alternative pathway. The reasons for a reduced complement opsonization due to LTA d-alanylation might be found in electrostatic interactions, as stronger binding of C3 to negatively charged lipid membranes in comparison with neutral or positively charged lipid membranes has been described (Yorulmaz et al., 2016). In addition, surfaces with amino groups in combination with hydrophobic CH_3_ groups (as it is found in alanine) might more strongly absorb serum proteins such as albumin, causing the formation of a protein layer that inhibits access of C3b (Toda and Iwata, 2010).

After opsonization of the streptococci with hyperimmune serum, we found high levels of association of *S. suis* wt and 10Δ*dltA* with monocytes but not with lymphocytes. In addition, the secretion of IL-1β and TNF-α strongly increased. This is likely due to a crosstalk between signaling of antibody-recognizing Fc receptors and PRR signaling in the monocytes associated with *S. suis*, as described for human dendritic cells exposed to opsonized *Staphylococcus aureus* (den Dunnen et al., 2012). IL-1β is produced by activated macrophages and monocytes, whereby the cleavage of pro-IL-1β to active IL-1β by caspase-1 is inflammasome-dependent (Martinon et al., 2002). Active inflammasomes also regulate gasdermin D activation resulting in pore formation and IL-1β release in the context of pyroptosis (Chen et al., 2020), which might account for the membrane damage we observed in monocytes after 2 h infection (Figure 4A). High local levels of the pore-forming toxin suilysin, produced by *S. suis*, might also be involved in this phenomenon as suilysin is known to induce IL-1β by inflammasome activation in murine macrophages (Song et al., 2020).

In difference to our finding of an augmented secretion of IL-1β and TNF-α in response to antibody-opsonized bacteria, Segura et al. (2006) described reduced mRNA expression of cytokines in a whole blood culture system with *S. suis* opsonized by specific antibodies, possibly due to suppressed bacterial growth. In our PBMC samples, bacteria proliferated even in the presence of high levels of specific antibodies (Supplementary Figure S8), most likely because there were no granulocytes present.

In contrast to the observed cytokine induction in isolated PBMCs, in whole porcine blood infected with *S. suis* 10Δ*dltA* for 2 h, we found IL-1β but not TNF-α levels to be reduced compared to blood infected with the wt (Figure 6; Supplementary Figure S4). This finding suggests a possible influence of LTA d-alanylation on inflammasome regulation, as TNF-α production and secretion are not inflammasome-dependent. Interestingly, direct recognition of cytosolic LTA of *Listeria monocytogenes* by the NLRP6 inflammasome was described for macrophages of mice (Hara et al., 2018), while the impact of d-alanylation was not investigated in this context. Since the reduction in IL-1β levels was only visible in whole blood, but not in isolated PBMCs, we speculate that a crosstalk between different immune cells in porcine blood might be important for this phenotype.

In summary, we found d-alanylation of LTAs in *S. suis* to be an important factor in CAMP resistance, defense against phagocytosis by granulocytes, reduction of complement deposition, and association with porcine monocytes and lymphocytes. Further studies are warranted to elucidate how *S. suis* modulates the host response through d-alanylation of its LTA and potentially also its WTA.

## Data Availability Statement

The original contributions presented in the study are included in the article/**Supplementary Material**, further inquiries can be directed to the corresponding author.

## Ethics Statement

The animal study was reviewed and approved by the state Saxony (Landesdirektion Sachsen), Germany, under the permit numbers TVV 57-18 and A09/19 (collection of blood from piglets).

## Author Contributions

SÖ designed and conducted all experiments except RNA analysis, LTA extraction and NMR spectroscopy. Furthermore, SÖ analyzed the data and drafted the manuscript. A-KK supported bactericidal assays. NG supervised LTA extraction and NMR spectroscopy and analyzed NMR data. NB and MM supported experiments investigating interaction and antimicrobial peptides and performed RNA analysis. NS supervised flow cytometry analysis. SÖ, CB, and MK-B conceived the study and designed experiments. All authors contributed to the article and approved the submitted version.

## Funding

This work was funded by the German Research Foundation grant BA 4730/4-1 and KO 3552/7-1 to CB and MK-B, respectively.

## Conflict of Interest

The authors declare that the research was conducted in the absence of any commercial or financial relationships that could be construed as a potential conflict of interest.

## Publisher’s Note

All claims expressed in this article are solely those of the authors and do not necessarily represent those of their affiliated organizations, or those of the publisher, the editors and the reviewers. Any product that may be evaluated in this article, or claim that may be made by its manufacturer, is not guaranteed or endorsed by the publisher.

## Footnotes

^1^ http://www.sanger.ac.uk
