# Supplementary material for: d-Alanylation of Lipoteichoic Acids in Streptococcus suis Reduces Association With Leukocytes in Porcine Blood
Source: Front Microbiol. 2022 Apr 18;13:822369. doi: 10.3389/fmicb.2022.822369 (PMC9058155; doi:10.3389/fmicb.2022.822369)
Supplement: Supplementary file 1 [file Data_Sheet_1.pdf]

Supplementary Material

D-Alanylation of Lipoteichoic Acids in *Streptococcus suis* Reduces Association with Leukocytes in Porcine Blood

S. Öhlmann[1], A.-K. Krieger[1], N. Gisch[2], M. Meurer[3,4], N. de Buhr[3,4], M. von Köckritz-Blickwede[3,4], N. Schütze[5], C.G. Baums[1]*

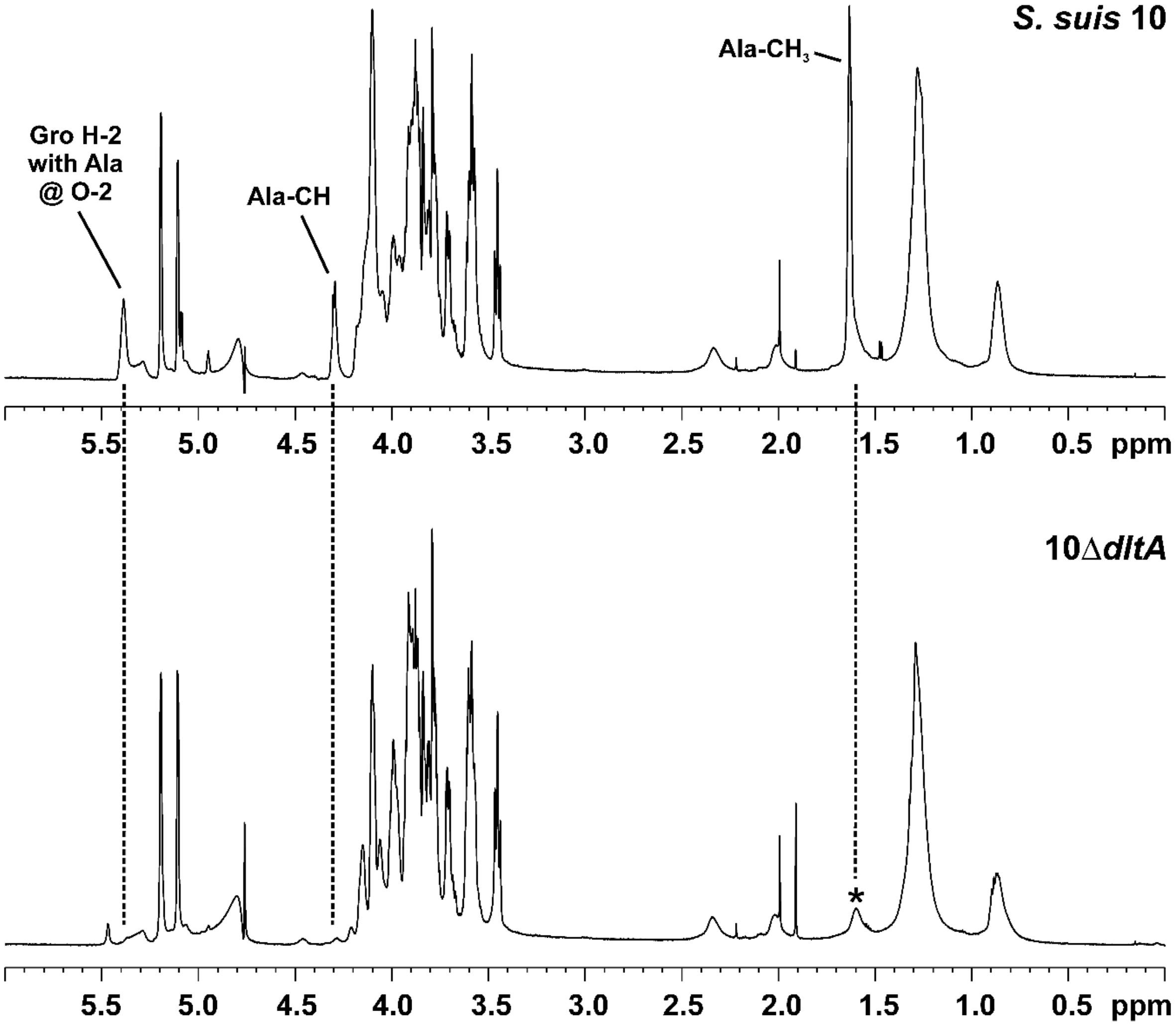

Figure S1: Lipoteichoic acids (LTA) of *S. suis* 10Δ*dltA* mutant does not possess alanine residues.

^{1}H Nuclear magnetic resonance spectroscopy of *S. suis* strain 10 (top panel) and 10Δ*dltA* mutant (bottom panel) LTA. The lines indicate the indicative peaks for D-alanine residues at the position O-2 of glycerol within poly-(glyco)glycerolphosphate chains, which are present in the spectrum recorded from LTA isolated from the wt but not in that of LTA isolated from the mutant. Residual signals at δ_H 1.65-1.60 ppm in spectra recorded from LTA of Δ*dltA* mutant (marked with *) represent the β-protons of fatty acids.

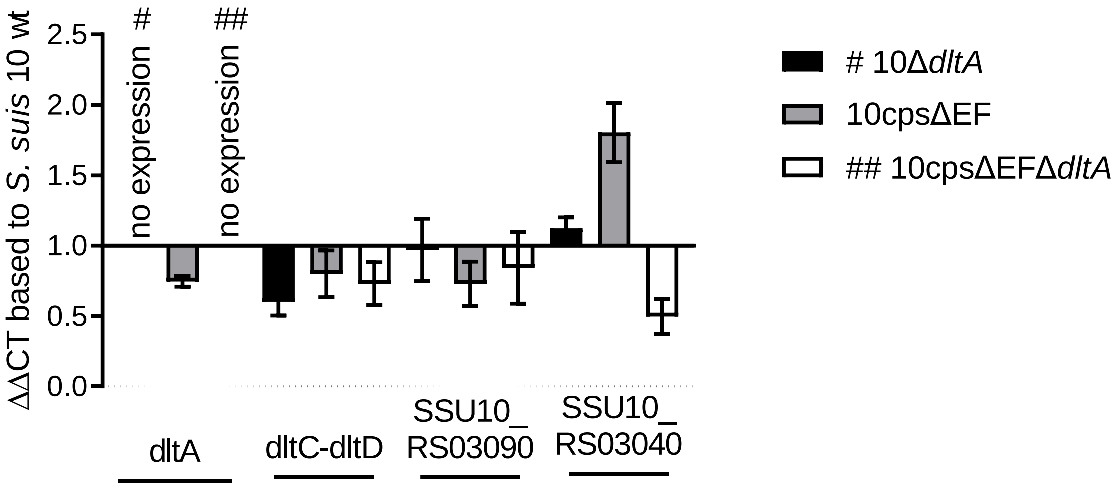

Figure S2: In frame deletion of *dltA* in 10Δ*dltA* does not affect expression of *dltC-dltD* nor of an ORF upstream (SSU10_RS03040, glucosamine-6-phosphate deaminase) or the ORF directly downstream of the *dlt* locus (SSU10_RS03090, low temperature requirement protein A).
RT-PCR was conducted with oligonucleotide primers listed in Table S1. Relative transcript levels of 10Δ*dltA* as well as the unencapsulated mutants 10cpsΔEF and 10cpsΔEFΔ*dltA* were compared to those of strain 10 wt by calculation of ΔΔCT using *gyrB* expression as reference. Additionally, we tested *dltA* RNA expression and did not measure any signal in the *dltA* mutants after 40 cycles. Data are presented as means ± SD of ΔΔCT of three independent experiments.

Table S1: Oligonucleotide primers used in qRT-PCR

Gene	Primer sequence (5´-3´) forward	Primer sequence (5´-3´) reverse	Amplicon length (bp)
gyrB	GGACCTGGGTGCTTAACAGA	AGGTGGTACCCATGAGCAAG	158
dltA	CCTGTCCTTGTTTTTGGCGG	CACCAAACTCGGCTCTGCTA	150
dltC-dltD	CATTTCTGGTGTTGTGGAGTTGA	ACCGAGAAGAACTCGGGAAAA	120
SSU10_ RS03090	ACGTTGCCAGCTGATTACCA	AGAGTCGCCATTGCGAAGAA	120
SSU10_ RS03040	TAGGTTTGGCAACAGGCTCC	TGGTCGCTCTCCTCACCTAA	127

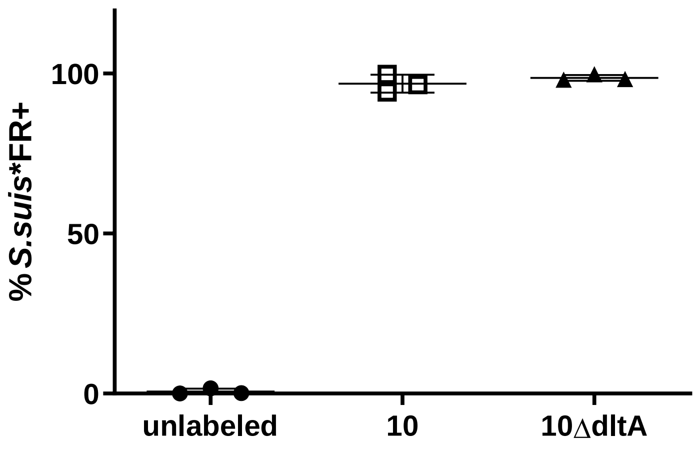

Figure S3: Comparison of Far Red labeling of *S. suis* 10 and 10Δ*dltA*.

Far Red labeled *S. suis* 10 wt and mutant strain were analyzed for the percentage of positively labeled bacteria by flow cytometry. Statistical analysis was performed by Mann Whitney test and no significant difference between wt and isogenic mutant was recorded.

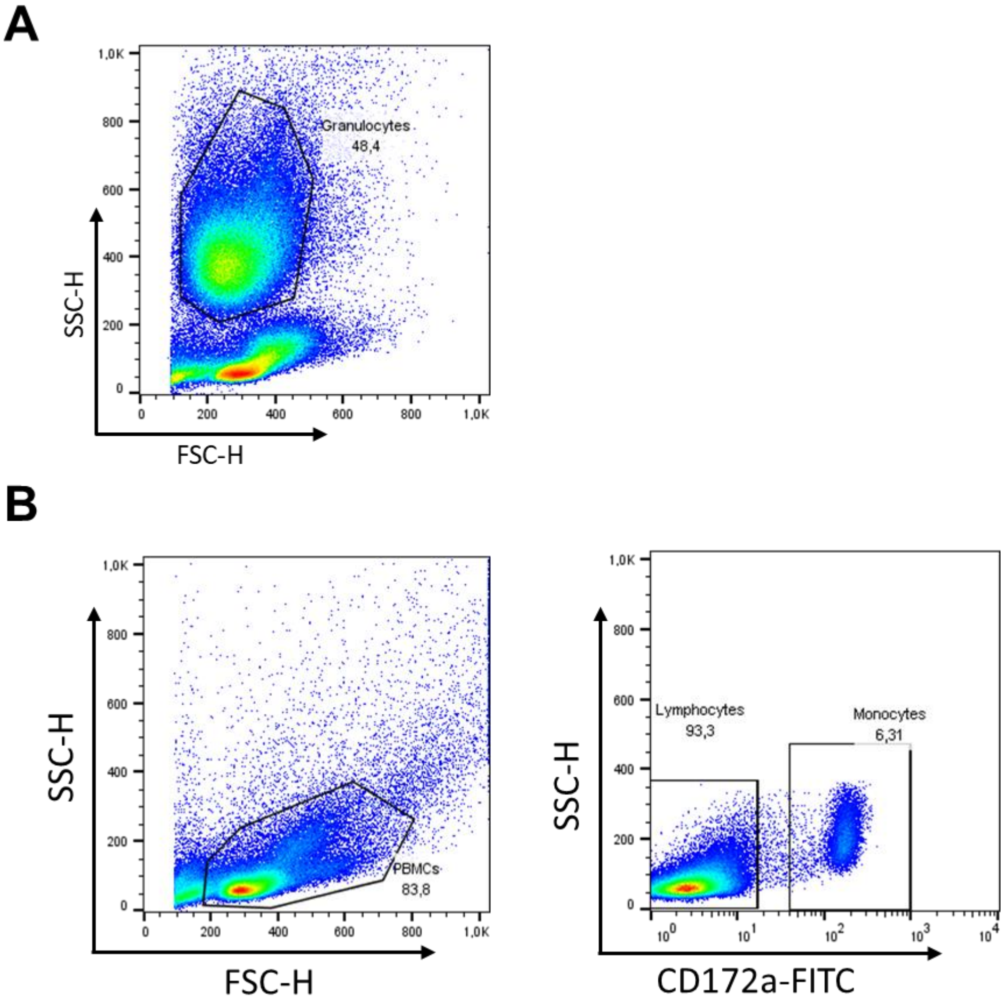

Figure S4: Gating strategy of granulocytes in whole blood (A) and monocytes in isolated PBMCs (B).

Flow cytometry measurement of granulocytes in whole blood was performed in assays depicted in Figure 2, while isolation of PBMCs and staining of monocytes with the FITC-labeled marker CD172a was conducted in experiments shown in Figure 3.

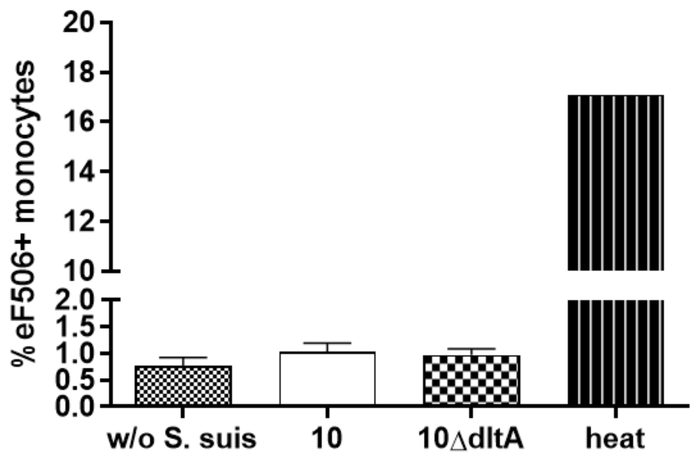

Figure S5: Viability staining of peripheral blood mononuclear cells (PBMCs) indicates no increased cytotoxicity towards monocytes of *S. suis* 10 or 10Δ*dltA* after 30 min of incubation.

PBMCs (1 x 10^7 /ml) were incubated with *S. suis* 10 wt or 10Δ*dltA*, that had been pre-incubated in serum of colostrum-deprived piglets (CDS). After 30 min viability dye eF506 was used to mark membrane damaged cells and monocytes were stained with FITC-coupled myeloid marker CD172a. Cells treated with PBS without bacteria (w/o *S. suis*) and heat treated (2 x 5 min at 56 °C) PBMCs were used as negative and positive control, respectively. Samples were analyzed by flow cytometry. Bars and error bars represent mean and SD of three biological replicates (heat treatment to verify eF506 staining was only performed with one sample). Statistical analysis was performed by Kruskal-Wallis and Dunn's multiple comparison test and no significant differences were detected between samples with or without *S. suis*.

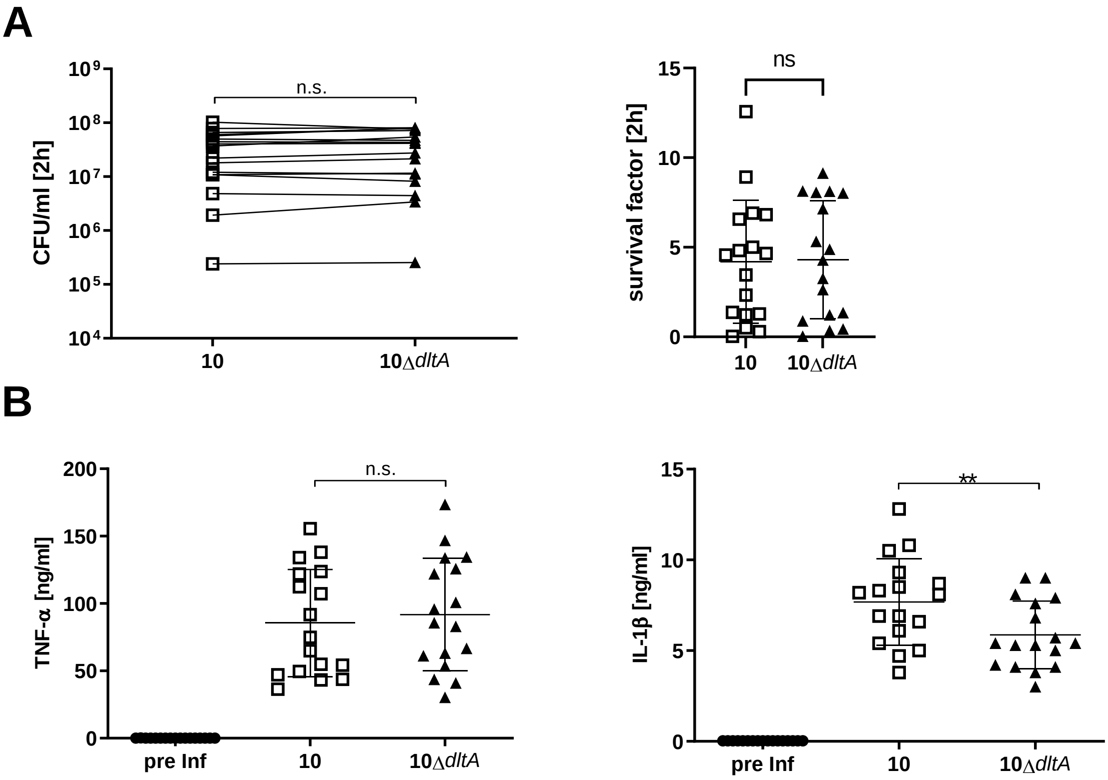

Figure S6: S. *suis* strain 10 induces significantly higher levels of IL-1β in porcine blood than the mutant 10Δ*dltA*.

Porcine plasma was obtained after incubation of blood with *S. suis* strain 10 or 10Δ*dltA* for 2h at 37 °C under constant rotation. CFU and survival data are shown in A. For B cytokine levels were determined by ELISA. Horizontal lines and error bars represent mean values and standard deviations, respectively. The limit of detection was 0.016 ng/ml for TNF-α and 0.031 ng/ml for IL-1β. Bars and error bars represent mean and SD. For statistical analysis Shapiro-Wilk normality test and paired t-tests between wt and mutant samples were performed. ** $p < 0.01$, not significant (n.s.) $p > 0.05$.

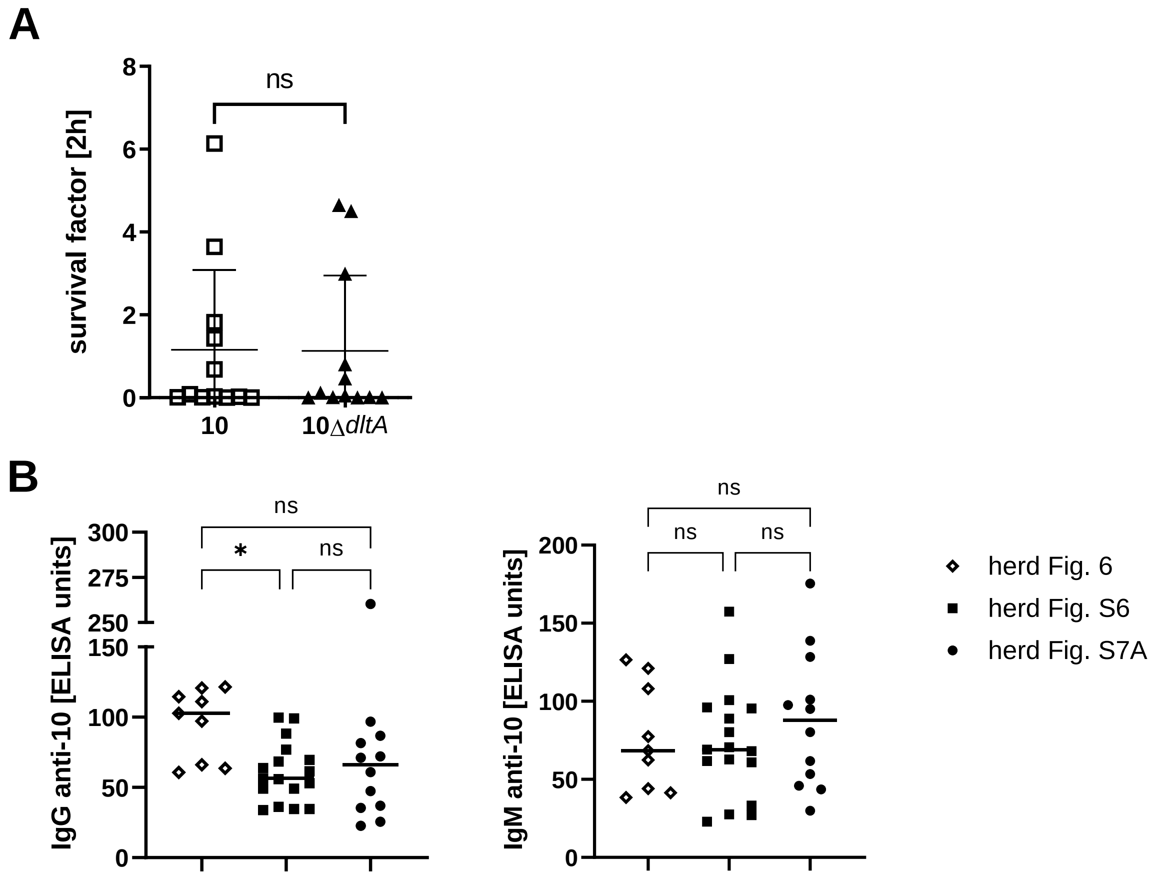

Figure S7: Comparison of survival of *S. suis* 10 wt and 10Δ*dltA* in whole blood obtained from piglets of a third conventional herd (A) and levels of plasma IgG and IgM antibodies binding to the surface of *S. suis* strain 10 present in the bactericidal assays shown in the indicated figures (blood samples collected in 3 different herds) (B).

A Whole porcine blood was infected with *S. suis* 10 and 10Δ*dltA* for 2 h at 37 °C under constant rotation. The survival factor was calculated by dividing the CFUs obtained after and before the 2h incubation period. **B** Levels of plasma IgG and IgM binding to the surface of inactivated and immobilized *S. suis* wt strain 10 as determined in ELISA. Plasma was obtained of all three indicated bactericidal assays (piglets from different herds for each assay) and stored at -80°C until measurement. A hyperimmune serum (#4515) obtained after prime-boost vaccination of a piglet with a *S. suis* strain 10 bacterin was used as a standard and defined to include 100 ELISA Units. Bars and error bars represent mean and SD. For statistical analysis Shapiro-Wilk normality test, Wilcoxon matched-pairs signed rank test between wt and mutant samples (**A**) as well as Kruskal-Wallis test followed by Dunn's multiple comparisons test (**B**) were performed. * $p < 0.05$, not significant (n.s.) $p > 0.05$.

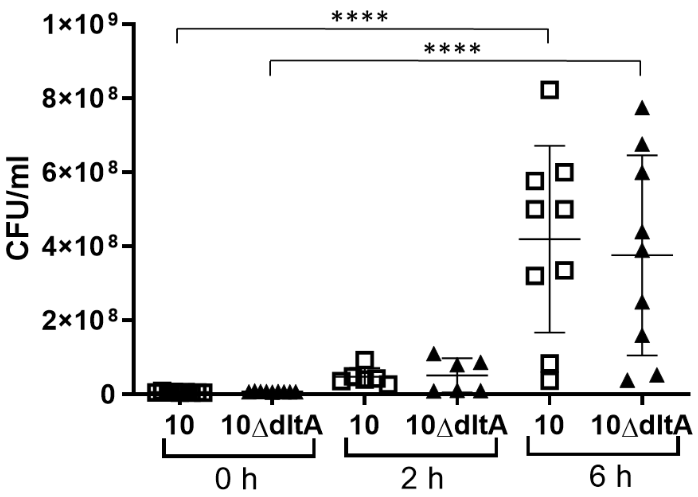

Figure S8: Colony forming units (CFU) in PBMC samples infected with *S. suis* 10 or 10Δ*dltA* for the indicated times after pre-opsonization in hyperimmune serum.

Serial dilution and plating on blood agar plates were conducted to determine CFU of *S. suis* 10 and 10Δ*dltA* in the PBMC samples used to evaluate cytokine production as shown in Fig. 5. Normality was tested using Shapiro-Wilk test. For statistical analysis Kruskal-Wallis test followed by Dunn's multiple comparisons test were performed. Differences that are not indicated are not significant. **** $p < 0.0001$
